# Tumor microenvironment remodeling and tumor therapy based on M2-like tumor associated macrophage-targeting nano-complexes

**DOI:** 10.7150/thno.50928

**Published:** 2021-01-01

**Authors:** Shulan Han, Wenjie Wang, Shengfang Wang, Tingyuan Yang, Guifeng Zhang, Di Wang, Ruijun Ju, Yu Lu, Huimei Wang, Lianyan Wang

**Affiliations:** 1 College of Chemistry, Chemical Engineering and Resource Utilization, Northeast Forestry University. Harbin 150040, P. R. China; 2 Key Laboratory of Green Process and Engineering, State Key Laboratory of Biochemical Engineering, Institute of Process Engineering, Chinese Academy of Sciences, Beijing 100190, P.R. China; 3 Beijing Institute of Petrochemical Technology, Beijing 102617, P.R. China; 4 Institute of Veterinary Immunology & Engineering, Jiangsu Academy of Agricultural Sciences, Nanjing 210014, Jiangsu, P.R. China

**Keywords:** nano-complex, tumor associated macrophage, tumor microenvironment, anti-tumor therapy

## Abstract

**Background:** Among the many immunosuppressive cells in the tumor microenvironment, tumor-associated-macrophages (TAMs) are well known to contribute to tumor development. TAMs can be conditioned (polarized) to transition between classical M1-like macrophages, or alternatively to M2-like macrophages. Both are regulated by signaling molecules in the microenvironment. M1-like TAMs can secrete classic inflammatory cytokines that kill tumors by promoting tumor cell necrosis and immune cell infiltration into the tumor microenvironment. In contrast, M2-like TAMs exhibit powerful tumor-promoting functions, including degradation of tumor extracellular matrix, destruction of basement membrane, promotion of angiogenesis, and recruitment of immunosuppressor cells, all of which further promote tumor progression and distal metastasis. Therefore, remodeling the tumor microenvironment by reversing the TAM phenotype will be favorable for tumor therapy, especially immunotherapy.

**Methods:** PLGA nanoparticles encapsulating baicalin and melanoma antigen Hgp peptide fragment 25-33 were fabricated using the ultrasonic double-emulsion technique. The nanoparticles were further loaded with CpG fragments and used conjugated M2pep and α-pep peptides on their surfaces to produce novel nano-complexes. The capability to target M2-like TAMs and anti-tumor immunotherapy effects of nano-complexes were evaluated by flow cytometry and confocal microscopy *in vitro*. We also investigated the survival and histopathology of murine melanoma models administrated with different nanocomplexes. Improvements in the tumor microenvironment for immune attack of melanoma-bearing mice were also assessed.

**Results:** The nano-complexes were effectively ingested by M2-like TAMs *in vitro* and* in vivo*, and the acidic lysosomal environment triggered the disintegration of polydopamine from the nanoparticle surface, which resulted in the release of the payloads. The released CpG played an important role in transforming the M2-like TAMs into the M1-like phenotype that further secreted inflammatory cytokines. The reversal of TAM released cytokines and gradually suppressed tumor angiogenesis, permitting the remodeling of the tumor microenvironment. Moreover, the activated TAMs also presented antigen to T cells, which further stimulated the antitumor immune response that inhibited tumor metastasis. Activated T cells released cytokines, which stimulated NK cell infiltration and directly resulted in killing tumor cells. The baicalin released by M1-like TAMs also killed tumor cells.

**Conclusion:** The nano-complexes facilitated baicalin, antigen, and immunostimulant delivery to M2-like TAMs, which polarized and reversed the M2-like TAM phenotype and remodeled the tumor microenvironment to allow killing of tumor cells.

## Introduction

Malignant tumors are the major killers currently threatening human health around the world with rising morbidity and mortality 1, 2. Tumor immunotherapy is an effective anti-tumor strategy that has been developed in recent years 3, 4; it has shown the capacity to activate a patient's own immune system, and then identify and eliminate tumors, which avoids much damage to normal cells. Tumor immunotherapy also inhibits tumor metastasis by improving systematic immunity 4. However, it is currently recognized that one of the toughest barriers to anti-tumor immunotherapy is immunosuppression created by the tumor microenvironment. Therefore, remodeling the microenvironment to promote immune cell infiltration, suppress tumor angiogenesis, and restrain tumor metastasis is very important for enhancing the anti-tumor effects of drugs. Tumor-associated macrophages (TAMs), especially M2-like TAMs, are an important type of strategic cell to promote immunosuppression and extend drug effectiveness in the tumor microenvironment 5-7. TAMs are designated as M1-like differentiated macrophages with antitumor properties, or as M2-like macrophages with tumor-supporting functions, depending on the cell polarization achieved by signaling molecules in the microenvironment 5, 8. M2-like macrophages play an important role in the formation of tumor-associated fibroblasts and invading endothelial cells by secreting matrix metalloproteinases (MMPs) 9. M2-like macrophages are also mainly responsible for producing angiogenic factors 10. Therefore, to promote effective tumor therapy it is crucial to reprogram the TAMs phenotype from M2-like to M1-like by remodeling the tumor microenvironment.

Most previous studies have used CpG-ODN as a common immune stimulator to transform the TAMs phenotype. Consequently, immunological responses of T helper cells (Th1) were enhanced by IL-12, IFN-γ, and TNF-α secreted by TAMs 11. Furthermore, some literature that attracted widespread attention reported that baicalin extracted from *Scutellaria baicalensis* Georgi exhibits dual functions of immune-modulation and select cytotoxicity 12-14. In our previous study, we reported that PLGA nanoparticles carrying a low dose of baicalin activate immune cells 13. In addition, Gong* et al.* reported that baicalin stimulates T and B cell proliferation, independently and cooperatively with concanavalin A (Con-A) or lipopolysaccharide (LPS) 15. Orzechowska *et al.* determined that the immune system modulation by baicalin in a patient with acute lymphocytic leukemia was due to increased production of IFN-γ in peripheral blood leukocytes 16. From the foregoing reports, we surmised that baicalin, as a natural multi-effect drug, offered a broad range of prospective applications for tumor immunotherapy. Therefore, the immuno-modulatory activity of baicalin was helpful in breaking the barrier of immunosuppression that might be favorable for promoting anti-tumor T cell mediated cytotoxicity. The combination of CpG-ODN and baicalin was expected to improve the immunosuppression of TAMs caused by the tumor's location and its stimulated micro-environment, which gives rise to the anti-tumor responses.

The main priority for reversing the TAMs phenotype is to effectively transport the immune stimulator to the affected TAMs. Therefore, it is necessary to develop a delivery system with high affinity target ligands to ensure effective distribution of the immune-stimulator to specific cells. Recently, some studies have reported that a peptide, designated as M2pep (an M2-like macrophage binding peptide), as well as another α-peptide (a scavenger receptor B type 1 (SR-B1) targeting peptide), possess greater specificity to M2-like TAMs than to other leukocytes 17-19. As such, it is reasonable to speculate that the macrophages, acting as antigen presenting cells (APCs), deliver antigen to T cells, thus further activating T cell immune response for anti-tumor immunotherapy 20, 21. The baicalin taken up by TAMs is released to the tumor sites, which kill tumor cells. As mentioned above, the TAMs targeted delivery strategy is expected to assist baicalin and CpG to execute the dual anti-tumor roles of both remodeling the tumor microenvironment and killing tumor cells.

**Scheme 1:**
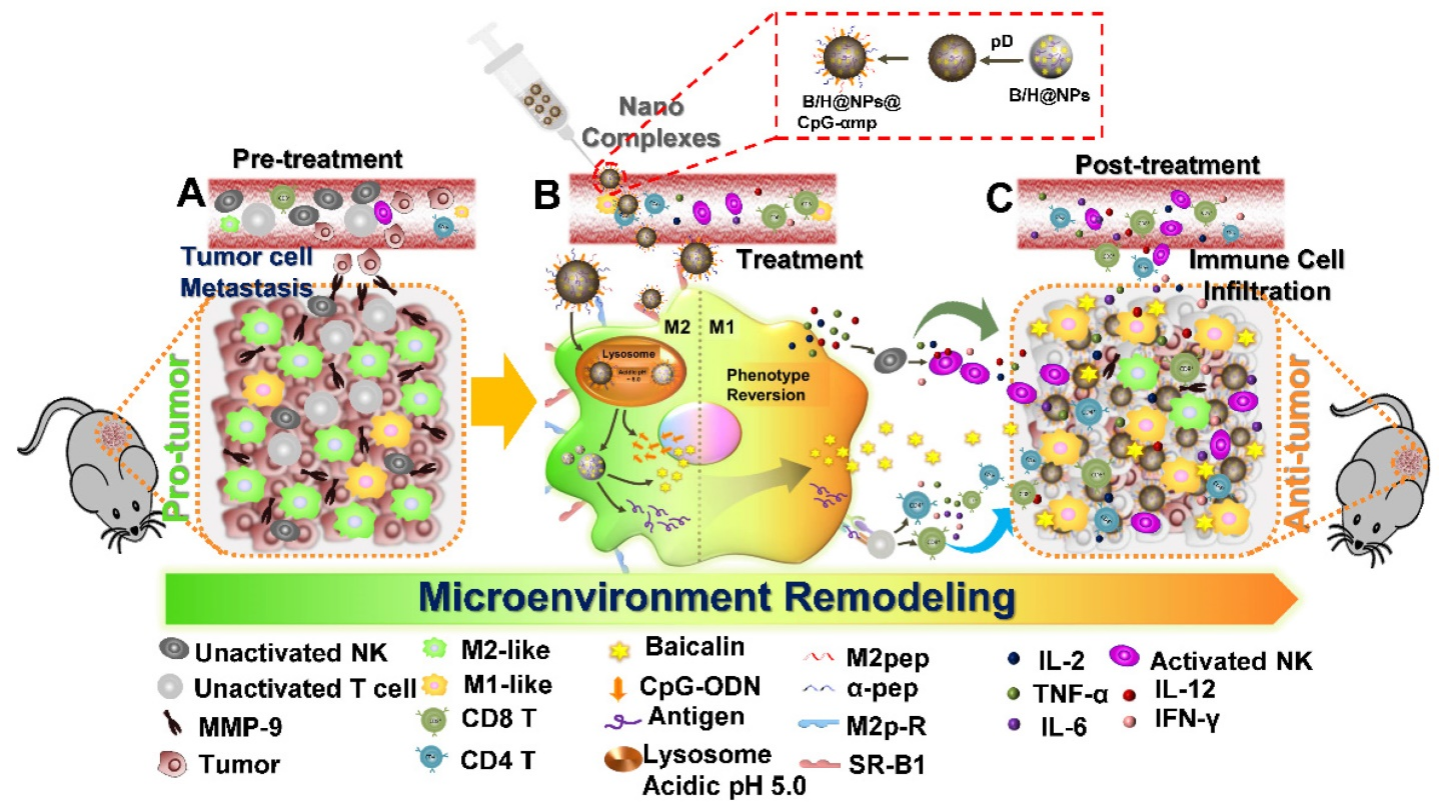
Schematic illustration of the action of nano-complexes in targeting M2-like macrophages for remodeling the tumor microenvironment. The scheme highlights the TAM phenotype reversal and tumor microenvironment transformation after treatment.

In this study, we successfully synthesized dual-targeting, M2-like TAMs nano-complexes by loading the anti-tumor drug and an immunostimulant to remodel the tumor microenvironment, as shown in Scheme 1. Baicalin was loaded in poly (lactic-co-glycolic acid) (PLGA) nanoparticles to overcome its low water-solubility and improve its systemic distribution and bioavailability. The tumor-associated antigen (Hgp100_25-33_) was simultaneously encapsulated in the PLGA nanoparticles, while CpG-ODN was adsorbed to a polydopamine (pD) coating layer on the nanoparticle surface. Polydopamine (pD) (a biomimetic of the specialized adhesive protein Mefp-5 [mytilus edulis foot protein-5] secreted from mussels) adheres to most material surfaces and permits conjugation of proteins and nucleic acids via a Michael addition or Schiff base reaction to its catechol functionalities 22. The nanoparticles were further coupled with M2pep and α-pep on their surfaces for dual targeting to M2-like TAMs. As shown in Scheme 1, there many immunosuppressive cells that reside in the pro-tumor microenvironment, including M2-like macrophages, un-activated NK cells, and T lymphocytes, all of which result in promoting an immunosuppressive microenvironment that assists tumor development (Scheme 1-A). In addition, M2-like macrophages and tumor cells secrete MMP-9 and VEGF, which can also promote tumor cell metastasis and development. In this study, the nano-complexes effectively target M2-like macrophages and deliver CpG and baicalin, which further reverses the transformation of M2-like macrophages to M1-like ones (Scheme 1-B). The mature and activated M1-like macrophages act as the second delivery carrier to present antigen that activates T cells. At the same time, certain cytokines, such as IL-2, IL-12, TNF-α, and IFN-γ, are released by activated M1-like macrophages and T cells. The cytokine activated NK cells then reduce their MMP-9 and VEGF secretions at tumor sites, which further transforms the pro-tumor microenvironment to an anti-tumor one. In addition, secondary delivery of baicalin by macrophages plays an anti-tumor chemotherapy role (Scheme 1-C). Here, we investigate the anti-tumor immune responses *in vitro* and *in vivo* using this collaborative strategy to remodel the tumor microenvironment and influence tumor therapy utilizing nano-complexes to target M2-like TAMs.

## Materials and reagents

Poly (D, L-lactic-co-glycolic acid) (75:25 PLGA, Resomer® RG 752H, M_W_ 4A) was purchased from Lakeshore Biomaterials (Birmingham, AL, USA). Baicalin (purity >95%) was purchased from Shanxi Anshun Biotechnology Co., Ltd. (Shanxi, China). Hgp100_25-33_ (KVPRNQDWL, melanoma antigen peptide, designated by Hgp), α-peptide (FAEKFKEAVKDYFAKFWD) and M2-peptide (YEQDPWGVKWWY) were synthesized by GL Biochem Ltd. (Shanghai, China). CpG (CpG 1826, B-class CpG ODN specific for mouse TLR9 macrophage, NK, and dendritic cell receptors) were synthesized by Sangon Biotech (Shanghai, China). Methylene chloride (AR grade) was purchased from Beijing Chemical Reagent Company (Beijing, China). Sodium cholate hydrate from ox or sheep bile (degree of purity ≥ 99%) was supplied by Sigma-Aldrich (St. Louis, Mo, USA). Methanol (GR grade), acetonitrile (GR grade), and phosphoric acid (AR grade) were purchased from Beijing Science Experiment Instrument Co., Ltd. (Beijing, China). Roswell Park Memorial Institute (RPMI) 1640 medium, Dulbecco's modified Eagle's medium (DMEM), and fetal bovine serum (FBS) were supplied by Gibco (Grand Island, NY, USA). Fluorochrome-labeled α-CD163, α-CD206, α-MHC II, α-CD86, α-F4/80, α-CD4, α-CD8a, α-CD3e, and α-NK1.1 anti-body for flow cytometry and mouse cytokine ELISA kits were purchased from eBioscience (San Diego, CA, USA). Antibodies for immunofluorescence staining were purchased from Abcam (Cambridge, UK).

### Preparation of PLGA nanoparticles loaded with baicalin and Hgp (B/H@NPs)

PLGA nanoparticles were assembled using an ultrasonic double-emulsion technique 13. First, 1 mL phosphate-buffered saline (PBS) (Gibco, Invitrogen Corp. CA, USA) containing 2 mg baicalin (B) and/or 3 mg Hgp100_25-33_ (H) were used as internal aqueous phase. Next, 5 mL methylene chloride containing 100 mg PLGA was employed as oil phase. The primary water in oil (W/O) emulsions were formed by adding the internal water phase to the oil phase under sonication (120 W; Digital Sonifier 450, Branson Ultrasonics Corp., Danbury, CT, USA) in a tube held within an ice bath for 40 s at the setting duty-cycle 50%, (4 s on, 2 s off). The primary emulsions were then added to 30 mL of the external aqueous phase containing 2% (w/v) sodium cholate to form a double emulsion suspension (W/O/W) after a second sonication in a tube held inside an ice bath for 2 min (duty-cycle 50%, 4 s on and 2 s off). Finally, the double emulsions were added to 45 mL of aqueous phase containing 0.5% w/v sodium cholate, which were further solidified into nanoparticles (H@NPs and B/H@NPs) by evaporating the dichloromethane under 450 rpm for 4 h at room temperature.

### Preparation of nano-complexes targeting M2-like TAMs (B/H@NPs@CpG-αmp)

PLGA nanoparticle surfaces were coated with polydopamine (pD) (PLGA-pD) to load CpG-ODN and targeting peptides. First, 5 mg baicalin/Hgp nanoparticles (B/H@NPs) were pre-incubated in 1 mL Tris-HCl buffer (0.01 M, pH 8.5) containing 2 mg dopamine hydrochloride (Sigma-Aldrich, St. Louis, MO, USA). The mixture was then incubated at room temperature for 5 h with rotation. Afterwards, the PLGA-pD NPs were collected by centrifugation (20000 g, 10 min), and suspended in fresh dopamine (Sigma-Aldrich) solution. They were then incubated with rotation for an additional 3 h at room temperature. The collected PLGA-pD NPs were next incubated in PBS (1 × 10^-3^ M, pH 6.8) with CpG (0.1% w/v) and rotated for 1 h at room temperature to prepare the nano-complexes of B/H@NPs@CpG. Finally, 50 µg M2pep (mp) and 50 µg α-peptide (α) were added to the nano-complexes suspension and rotated for 2 h at room temperature to obtain the nano-complexes that target M2-like TAMs (B/H@NPs@CpG-αmp). The composition details of each group of nanoparticles is shown in Table S1 of the Supporting Information.

### Drug-loading efficiency

The baicalin and Hgp encapsulated in PLGA-nanoparticles were measured. Five mg of freeze-dried NPs were incubated in 1 mL acetonitrile at room temperature for 2 min. After the nanoparticles were completely dissolved, the supernatant was used for assaying amounts of baicalin and Hgp by high-pressure liquid chromatography (HPLC). The drug-loading efficiency (LE) and encapsulation efficiency (EE) of baicalin and Hgp in nanoparticles were calculated as follows:

*LE (%) = (amount of drug in NPs/Weight of NPs) × 100*

*EE (%) = (Actual amount of drug encapsulated in NPs/Accrual amount of drug added to system) × 100*

The amounts of CpG loaded on the nanoparticles were analyzed using an Infinite 200 PRO NanoQuant plate (Tecan, Switzerland).

### Characterization of nano-complexes

The average size and polydispersity index (PDI) of nanoparticles were measured by a Nano-ZS Zeta Sizer (Malvern Instruments Ltd., Malvern, UK) after resuspending them in deionized water. The morphology of the nanoparticles was characterized using scanning electron microscopy (JEM-6700F, JEOL Ltd., Tokyo, Japan). The structures of B/H@NPs@CpG and B/H@NPs@CpG-αmp complexes were characterized via transmission electron microscopy (TEM, JEM-1230, Japan) at an accelerating voltage of 200 keV. The B/H@NPs@CpG-αmp stability in PBS was monitored by dynamic light scattering (DLS). The release of the drug from the nano-complexes for the case of B/H@NPs@CpG-αmp in PBS (pH 6.5 and pH 7.4) after 7 days was measured by HPLC and a microplate reader.

### Generation of bone marrow-derived macrophages (BMDMs)

BMDMs were cultured from bone-marrow cells according to an established protocol 17. In brief, bone marrow (BM) cells were harvested from the femur and tibia bones of 6 to 8 weeks-old C57BL/6 mice. Bone marrow (BM) cells were then cultured in RPMI medium 1640 supplemented with macrophage colony stimulating factor (M-CSF, ThermoFisher, 20 ng/mL) for 6 days at 37°C to harvest immature BMDMs. On day 5, immature macrophages were cultured further for 48 h in new medium with different cytokines as follows: IFN-γ (ThermoFisher, 20 ng/mL) and LPS (100 ng/mL) for M1-like macrophages, and IL-4 (ThermoFisher, 20ng/mL) for M2-like macrophages. The harvested macrophages were employed for *in vitro* experiments of cellular function.

### Qualitative evaluation of nano-complexes uptake by confocal laser scanning microscopy (CLSM)

For the cellular uptake study, macrophages were incubated with the nano-complexes of B/H@NPs@CpG and B/H@NPs@CpG-αmp. First, macrophages were seeded in 20 mm glass-bottom dishes at a density of 5 × 10^4^ cells in 1 mL of culture medium. Second, Nile Red- labelled nano-complexes were added and co-cultured with macrophages for 12 h. Finally, the nano-complexes were taken up into macrophages and observed by CLSM (Leica TCS SPS). The group receiving αmp-blocking was pretreated by administrating saturating concentrations of M2pep and α-peptide. The treated macrophages were fixed in 100 μL 4% paraformaldehyde for 15 min. The cyto-membranes and cell nuclei were then labeled with Alexa Fluor 488-conjugated Phalloidin and DAPI for 20 min, respectively. The samples were observed and imaged by CLSM after washing twice with PBS to remove unbound dye.

### Quantitative evaluation of nano-complexes uptake by flow cytometry

Flow cytometry (Beckman Coulter CyAn ADP) was used to further quantify the cellular uptake of the B/H@NPs@CpG and B/H@NPs@CpG-αmp nano-complexes. First, the macrophages were seeded in 24-well plates for 12 h, allowing cell attachment to occur. After cells reached 70-80% confluence, they were incubated with Nile Red-loaded nanoparticles for 12 h. The group receiving M2pep-blocking was pretreated by high concentrations of M2pep and α-peptides. Finally, the treated macrophages were washed with PBS to remove excess nano-complexes for analysis by flow cytometry and the mean fluorescence intensity of each cell was measured.

### Cellular cytotoxicity of macrophages

Cell viability was assessed using a colorimetric Cell Counting Kit-8 (CCK-8) assay (Dojindo laboratories, Kyoto, Japan), Cells were plated at a concentration of 2 × 10^4^ cells in 96-well plates containing 200 µL growth medium per well. Subsequently, the macrophages were co-cultured with 100 µg aliquots of various nano-complexes for 24 h. Ten µL CCK-8 reagent was then added to each well and further incubated with cells at 37°C for 4 h. Absorbance at 450 nm was measured and the viability results were calculated from the ratio between the average OD450 values of wells containing NP-stimulated cells to the counterparts containing unstimulated cells with medium.

### *In vitro* evaluation of anti-tumor effect of nano-complexes by flow cytometry

B16 cells were cultured in DMEM supplemented with 10% FBS at 37°C in a humidified atmosphere containing 5% CO_2_. The melanoma cells were then seeded in 24-well plates and treated with various nano-complex formulations for 24 h, identified as PBS, Hgp, H@NPs, B/H@NPs, B/H@NPs@CpG, and B/H@NPs@CpG-αmp. Cells were collected after trypsin digestion and centrifuged at 500 × g for 5 min. Ten µL Annexin V-FITC conjugate and 10 µL propidium iodide (PI) were added to each tube and the cells were incubated for an additional 15 min at room temperature in the dark. Finally, the viability and apoptosis levels of cells were determined by flow cytometry.

### Apoptosis of tumor cells induced by BMDMs combined with nano-complexes *in vitro*

Various macrophage phases, such as M0, M1, and M2 types, were induced in RPMI medium 1640 supplemented with different cytokines as follows: M-CSF (20 ng/mL) to induce M0 macrophages; IFN-γ (20 ng/mL) and LPS (100 ng/mL) for induction of M1 macrophages; and IL-4 (20 ng/mL) for induction of M2 macrophages. The various macrophage phenotypes (M0, M1, M2 and the mixed macrophages) were then seeded in 12-well Trans well plates (upper compartment) and cultured for 12 h in the same medium. B16 cells were seeded in the lower compartment of each Trans well plate for 12 h. Then, nano-complexes targeting M2-like macrophages (B/H@NPs@CpG-αmp) were incubated with different phenotypic macrophages for 12 h. Finally, T cells were added to the upper compartment of the Trans well plate for 24 h to mimic immune cells with macrophages in the microenvironment. B16 cells in the lower compartment were then collected after trypsin digestion and centrifuged at 500 × g for 5 min. Ten µL Annexin V-FITC and 10 µL PI were subsequently added to each tube for fluorescence labeling. The cells were further incubated for an additional 15 min at room temperature in the dark. Finally, the melanoma cells were centrifuged and washed as preparation for determining cell viability and apoptosis by flow cytometry.

### Measurement of baicalin loading and unloading in macrophages with time

All macrophages were detached and removed by scrapping, washed in pH 6.5 PBS, and then counted. After spinning at 1500 rpm for 5 min, 4 million cells were re-suspended in 1 mL baicalin solution (PBS, 600 µg/mL) for 10 min. Cells were then separated from the baicalin solution by centrifugation and re-suspended in 7 mL DMEM containing 10% FBS. Tubes containing the cells were placed in a swing bucket at 37°C and centrifuged at 100 rpm. Baicalin incubated cells were extracted after 0.5 h, 1 h, 1.5 h, 2 h, 2.5 h, 3 h and 6 h intervals of incubation. Drug concentrations remaining in the media were determined by HPLC. Media was completely replaced by fresh media between each sample extraction. Baicalin solution concentrations before and after incubation with cells were also measured to determine the total amounts of drug loading and unloading in each time interval.

### *In vivo* tumor targeting of M2-like macrophage-targeting nano-complexes evaluated by immunofluorescence staining and flow cytometry

C57BL/6 mice (6-8 weeks old) were purchased from Vital River Laboratories (Beijing, China) and used for *in vivo* experiments. Melanoma implants (B16) were injected subcutaneously in the right armpit of mice and raised for two weeks. The melanoma tumor-bearing mice were given 500 μg Nile Red-loaded nanoparticles (B/H@NPs@CpG and B/H@NPs@CpG-αmp) via intravenous injection (*iv.*). Tumor tissues were then isolated from tumor-bearing mice at 6 h, 12 h, and 24 h after grafting. For immunofluorescence analysis, tumor tissues were fixed in 4% paraformaldehyde for 12 h at 4°C and then dehydrated in 30% sucrose solution. The tissues were then frozen in Tissue Tek-OTC^®^ (Sakura, Torrance, CA, USA) compound and sectioned in 8 µm slices using a freezing microtome (Leica, Germany). The sections were immuno-stained with FITC anti-mouse CD206 (Abcam, Britain) for identification of M2-like TAMs. At the end of processing, the sections were used to measure the co-localization of nanoparticles and M2-like TAMs by means of an automatic multispectral imaging system (PerkinElmer Vectra II). Other harvested tumor tissue extracts were ground into cell suspensions for further analysis by flow cytometry.

### Evaluation of *in vivo* anti-tumor efficacy of various nano-complexes

To investigate the anti-tumor efficacy of different formulations, tumor-bearing mice were treated with nano-complexes when the average tumor volume grew to approximately 100 mm^3^ (at day 8). The mice were randomly divided in six groups (for each group, n = 9) and intravenously injected with PBS, Hgp, H@NPs, B/H@NPs, B/H@NPs@CpG, and B/H@NPs@CpG-αmp complexes for seven contiguous days (500 μg/100 μL preparations administered each day, including CpG (5 μg), baicalin (3.9 μg), and Hgp (4.8 μg)). Relative tumor volumes (*V*) were measured every other day. All tumors were excised on day 18 after implantation, and the tumor inhibition rate (R) was calculated as follows:

*V = length × (width)^2^ / 2*

*R = (tumor volumes of control group - tumor volumes of treated group) / tumor volumes of control group × 100%*

To investigate the immunization efficacy of the nano-complexes against melanoma, the above nano-complexes were used to trigger macrophage activation in normal C57 mice on a continuous daily schedule for one week (7 times, 100 μL at one time, including CpG (5 μg), baicalin (3.9 μg), and Hgp (4.8 μg)). One day after the last vaccination, the mice were inoculated subcutaneously with 5 × 10^5^ B16 melanoma cells. Relative tumor volumes (*V*) were measured every two days. All tumors were excised on day 18 after implantation.

### Evaluation of cytokine levels

Cytokine levels in serum and at tumor sites were quantified using mouse α-IL-10, α-IL-12p70, α-IFN-γ, α-IL-6, α-IL-2 and α-TNF-α ELISA kits following the manufacturer's protocol (eBioscience). Raw data were calculated using a five-parameter curve obtained from the absorbance values of standards provided by the manufacturer.

### Anti-tumor immune responses by immunofluorescence staining

Tumor cell suspensions from six mice were prepared, and TAMs markers (CD163, CD206, CD86, and MHC II) were identified by flow cytometric analysis for evaluation of TAMs phase reversion. In addition, T-lymphocyte markers (CD4 and CD8) and NK cell marker (NK1.1) were used to identify T-lymphocyte and NK cell infiltration in the tumor microenvironment. Tumor tissues from three animals of each group were frozen for immunofluorescence staining. Immunofluorescence analysis was used to evaluate TAMs reversion, together with T-lymphocyte and NK cells infiltration. The frozen sections were immuno-stained with FITC anti-mouse CD206 (Abcam) and with Rhodamine-labeled anti-mouse CD86 for TAM reversion. FITC tagged anti-mouse IFN-γ, anti-mouse CD8, and anti-mouse NK, were used to identify immune cells infiltration measurements. In addition, the frozen sections were antibody stained using anti-mouse casepase-3, α-CD31, α-VEGF, and α-MMP9 for assessing tumor cell apoptosis and metastasis. After processing, all stained sections were measured by the automatic multispectral imaging system (PerkinElmer Vectra II). The immunofluorescence analysis was used to evaluate whether the tumor microenvironment was remodeled by M2-like TAMs reversion and infiltration of T and NK cells.

### Histopathological analysis

On the 18^th^ day after tumor seeding, tissues were collected from the transplant and analyzed using hematoxylin and eosin (HE) staining. Hearts, livers, spleens, lungs, and kidneys were harvested from tumor-bearing mice and fixed in 4% paraformaldehyde solution. The organs were embedded in paraffin, sectioned, and processed for HE staining. The sections were measured by an automatic multispectral imaging system (PerkinElmer Vectra II).

### Animal care

All animal experiments were performed in compliance with the guide for care and use of laboratory animals. The animal protocol was approved by the Institutional Animal Care and Use Committees at Institute of Process Engineering, Chinese Academy of Sciences.

### Statistical analysis

All results are expressed as the mean ± standard error of the mean (SEM). Statistical analysis was performed using GraphPad Prism 5.0 software (San Diego, CA, USA). Differences between two groups were tested using an unpaired, two-tailed, Student's *t*-test. Differences among multiple groups were tested with one-way ANOVA followed by Tukey's multiple comparison. Significant differences between groups are expressed as follows: *P < 0.05, **P < 0.01, or ***P < 0.001.

## Results and Discussion

### Characterizations of M2-like macrophage-targeting nano-complexes

**Figure 1:**
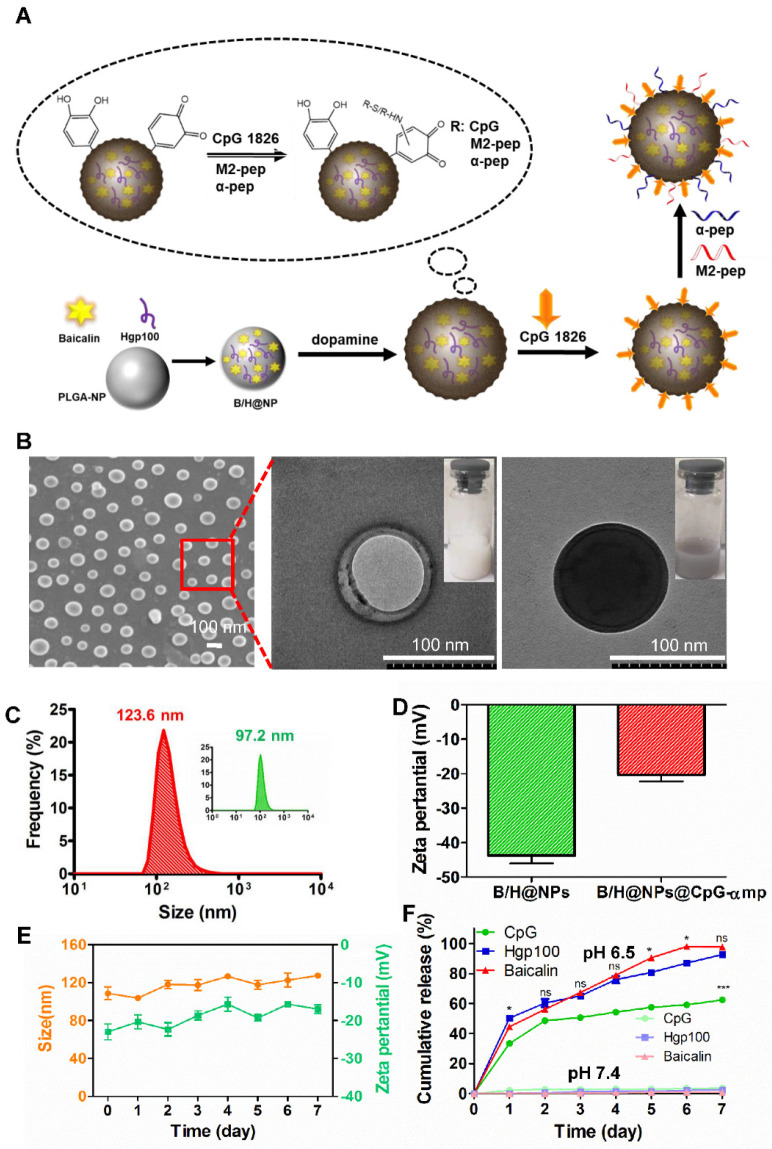
Preparation and characterization of the PLGA nano-complexes. (A) Schematic illustration of the PLGA nano-complexes preparation steps. (B) SEM and TEM images of the nano-complexes: NPs and pD@NPs, scale bar = 100 nm. (C) Size distributions of the nano-complexes: NPs and pD@NPs in pH 7.4 PBS. (D) Zeta potential of nano-complexes: NPs and pD@NPs in pH 7.4 PBS. (E) Variations of average size and zeta potential of nano-complexes: B/H@NPs@CpG-αmp in pH 7.4 PBS over 7 days. (F) Payload release profiles of nano-complexes: B/H@NPs@CpG-αmp in pH 6.5 and pH 7.4 PBS at 37°C over 7 days. The “*” is for Baicalin *vs* Hpg100; the “ns” is for Baicalin *vs* Hpg100, and the “***” is for Baicalin *vs* CpG, or Hpg100 *vs* CpG. Data are expressed as the mean ± standard error of the mean (SEM), n = 3. Differences between two groups were tested using an unpaired, two-tailed Student's *t*-test. Differences among multiple groups were tested with one-way ANOVA followed by Tukey's multiple comparison. Significant differences between groups are expressed as follows: *P < 0.05, **P < 0.01, or ***P < 0.001.

The nano-complexes containing baicalin and/or Hgp antigen (H@NPs and B/H@NPs) were fabricated using a double-emulsion technique combined with solvent evaporation. In Figure 1A, the drugs (baicalin and/or Hgp) and PLGA were assembled to form a stable drug-loaded nanoparticle structure. The nanoparticles were then incubated with dopamine under oxidative conditions (pH 8.5) to promote dopamine polymerization on the surface of nanoparticles. The obtained NPs turned black, indicating successful dopamine coating on the surface. CpG was subsequently incorporated on the surface coating layer of poly-dopamine (pD) via Michael addition or a Schiff base coupling reaction (B/H@NPs@CpG). The Tecan NanoQuant Plate analysis indicated that CpG was successfully loaded on the surface of pD-coupled B/H@NPs. The CpG-loading amounts on nanoparticles were found to be 11.2 µg/mg. After that, the M2pep (mp), and α-peptide (α-pep) were also conjugated to the surface of B/H@NPs@CpG under alkaline conditions, converting it to B/H@NPs@CpG-αmp. The whole preparation process was simple and mild, which avoided strong organic reagents and exhaustive purification steps.

The amounts of baicalin and Hgp encapsulated within the formulations were determined using high-performance liquid chromatography (HPLC). The encapsulation efficiency for baicalin and Hgp was 44.3 wt% and 30.2 wt%, respectively (Figure S1A, B). The drug loading amounts were 7.85 μg/mg for baicalin and 9.06 μg/mg for Hgp, which is expected to be sufficient to elicit cell-mediated immunity against tumors. The amounts of M2pep and α-peptide carried on the nanoparticles were also determined by the HPLC technique (Figure S1C, D). The combined nano-complex with one milligram carried 16.3 µg α-peptide and 18.9 µg M2pep. The loading efficiency reached 92% and 89% for the α-peptide and M2pep, respectively.

The structures of the nano-complexes including B/H@NPs and B/H@NPs@CpG-αmp were characterized by scanning electron microscopy (SEM) and transmission electron microscopy (TEM). A spherical core-shell structure was observed in the image of B/H@NPs (Figure 1B) as expected, indicating a successful encapsulation of water phase containing baicalin and Hgp. The third image in Figure 1B indicates that the B/H@NPs@CpG-αmp nano-complexes turned black after polymerization of the pD coating in comparison with nude B/H@NPs. The particle size of the combined nano-complexes was measured using dynamic light scattering (DLS). It was found that the mean of B/H@NP diameters was approximately 97.2 nm (Figure 1C, green curve), with an excellent polydispersity index (PDI) of 0.102. The particle size of B/H@NPs@CpG-αmp was found to be 123.6 nm (Figure 1C, red curve) with a PDI of 0.108. Compared with B/H@NPs, the size of B/H@NPs@CpG-αmp increased by 26.4 nm, which suggests the successful incorporation of polydopamine on the nanoparticle surface. In contrast to B/H@NPs, the zeta potential of the B/H@NPs@CpG-αmp increased from -43.1 ± 0.4 mV to -17.8 ± 0.3 mV (Figure 1D).

The B/H@NPs@CpG-αmp nano-complexes were suspended in phosphate-buffered saline (PBS, pH 7.4) to evaluate their stability. The particle size was continuously monitored via DLS for 7 days. The nanoparticles surface zeta was measured by a Nano-ZS Zeta Sizer also for 7 days under the same conditions. The zeta potential (green line) of B/H@NPs@CpG-αmp increased from -17.8 ± 0.3 mV to -16.2 ± 0.4 mV, and the size (orange line) slightly increased from 117.6 ± 0.6 nm to 128.8 ± 0.7 nm, as shown in Figure 1E. There was little change in zeta potential and size for the nano-complexes, indicating their high stability in PBS.

The *in vitro* release of baicalin, Hgp, and CpG from the nano-complexes was investigated by resuspending nano-complexes in PBS at pH 6.5 (close to the pH of the tumor microenvironment). There were two stages in the release curves. As seen in Figure 1F (green line), CpG exhibited an initial burst release during the first 48 hours due to the dissociation of polydopamine under acidic conditions. Subsequently, baicalin and Hgp were released from nano-complexes. The release of baicalin and Hgp was sustained over 7 days. For the first two days, Hgp release slightly exceeded baicalin, likely because it is a small hydrophilic molecule with high diffusivity (Figure 1F, blue and red line). Simultaneously, the *in vitro* release of baicalin, Hgp, and CpG from nano-complexes was investigated in PBS at pH 7.4 (close to the pH of the humoral microenvironment). Our results, as shown in Figure 1F, find that only a small amount of payload is released, and the highest cumulative release of CPG, baicalin, and Hgp maximized at 3.54%, 0.98% and 1.99% after 7 days, respectively. This indicates that the nano-complexes are highly stable in a body fluid environment, a necessary condition for the long circulation time needed to target tumors *in vivo*. The above results demonstrate that the nano-complexes designed in this study can be effectively loaded with baicalin, antigen, and CpG; exhibit good storage stability in PBS; and undergo slow release of payload in an acidic environment. The data suggest that the nano-complexes should achieve extended-release of payloads in a weak acid, tumor environment. These properties give support to our belief that these formulations will be found effective in countering the phenotype reversal in macrophage differentiation and thereby lead to prolonged anti-tumor responses.

### M2pep/α-peptide coupling to nano-complexes for targeting M2-like macrophages

In order to remodel the tumor microenvironment by reprogramming M2-like TAMs using nano-complexes, it was crucial to transport the payload to M2-like TAMs as a first step before its internalization. Recently, M2pep (mp) and α-peptide (α) were reported to be specific ligands for targeting M2-like TAMs 17. For the PLGA nanoparticles, their application was limited due to the low loading capacity because of fewer coupling groups. Here, we developed a novel polydopamine coating method to conjugate a greater mass of M2pep and α-peptide on the nanoparticle surface. The coupling efficiency significantly improved to 89% for M2pep (mp) and α-peptide (αp) via Michael addition and Schiff base reactions. We then tested the targeting capacity of B/H@NPs@CpG-αmp for M2-like TAMs. Bone marrow-derived macrophages (BMDMs) were cultured and converted to either the M1-like, or M2-like phenotypes (shown in Figure S2A). The M2-like macrophages were collected and assayed for uptake of nano-complexes with different targeting ligands (specifically, NPs@αp, NPs@mp, and NPs@αmp). As shown in Figure S2C-D, the NPs@αmp coupling with dual ligands displayed remarkably greater uptake than either of the single ligand carriers NPs@αp, or NPs@mp. These results indicate that the nanoparticles carrying dual targeting peptides demonstrate more specific and stronger targeting capability than those carrying a single targeting peptide. To verify the effectiveness of double peptide targeting, we performed additional experiments to determine whether soluble M2pep or soluble α-pep could preferentially bind to M2-like macrophages over M1-like macrophages. The result in Figure S3 shows clear evidence, from confocal images, that there is much more M2pep and α-pep bound to M2-like macrophages than to M1-like ones (Figure S3A-B). Little M2pep bound to M1-like macrophages. However, there is still a small amount of α-pep binding with M1-like macrophages (Figure S3A, C). α-pep is a scavenger receptor B type 1 (SR-B1) targeting peptide which also is expressed to a small extent on M1-like macrophages. They therefore bind a modest amount of α-pep (as shown in Figure S3B-C). According to the results above, we postulate that the double density of targeting peptides combines with M2-like macrophages more effectively than with M1-like macrophages.

**Figure 2:**
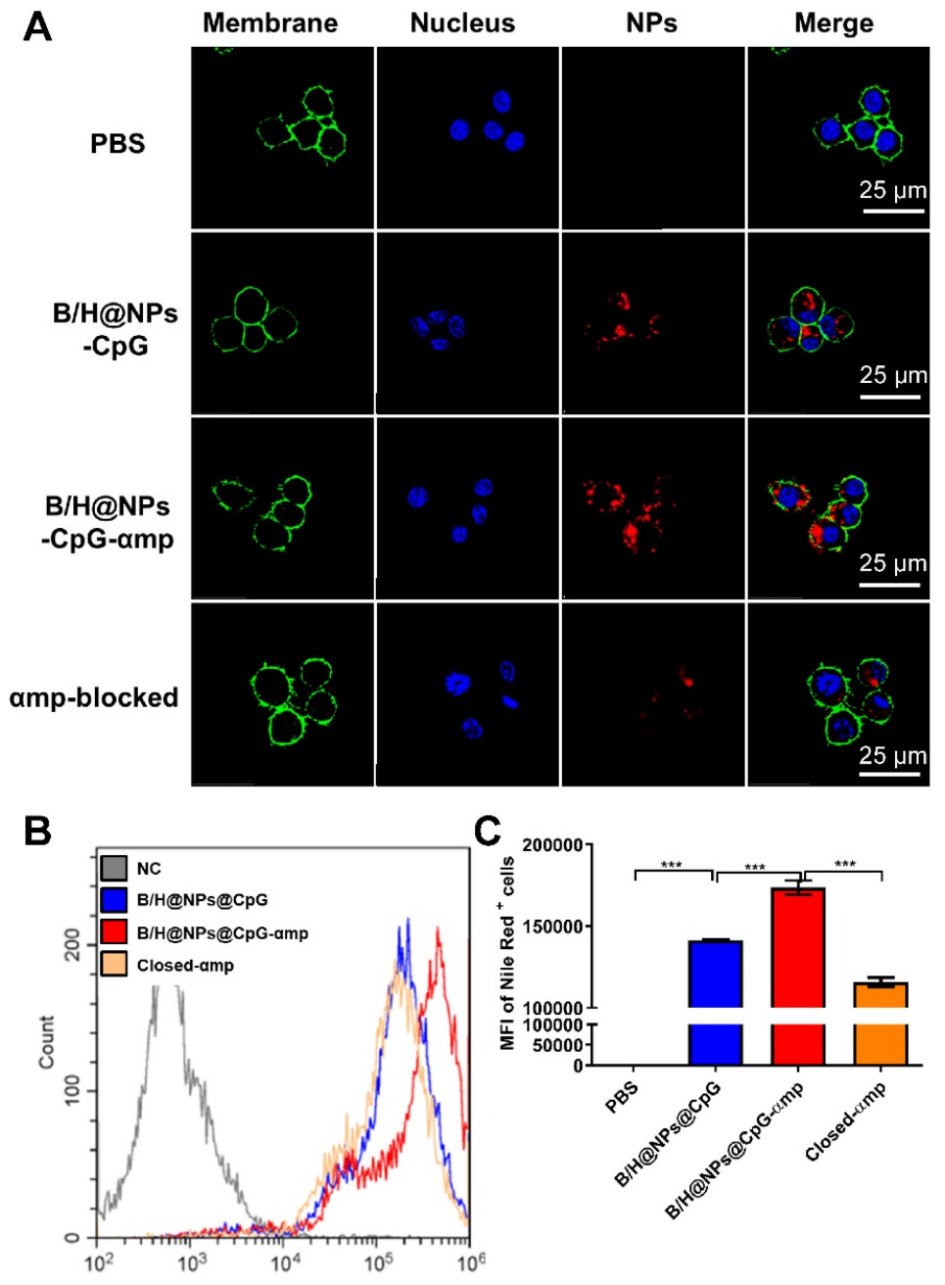
Nano-complexes targeting capacity to M2-like macrophages *in vitro* determined by confocal imaging and flow cytometry. (A) The M2-like macrophage targeting capability of the nano-complexes: B/H@NPs@CpG, B/H@NPs@CpG-αmp, and αmp-blocked was analyzed by confocal microscopy, scale bar = 25 μm. (B, C) The M2-like macrophage targeting capability of the nano-complexes B/H@NPs@CpG, B/H@NPs@CpG-αmp, and αmp-blocked was analyzed by flow cytometry. The group of αmp-blocked samples received pretreatment by administrating saturating concentrations of M2pep and α-peptide in advance of the administration of nano-complexes. Data are expressed as the mean ± standard error of the mean (SEM), n = 3. Differences between two groups were tested using an unpaired, two-tailed Student's *t*-test. Differences among multiple groups were tested with one-way ANOVA followed by Tukey's multiple comparison. Significant differences between groups are expressed as follows: *P < 0.05, **P < 0.01, or ***P < 0.001.

Accordingly, we selected the nano-complexes (B/H@NPs@CpG-αmp) containing dual targeting peptides for evaluation of M2-like macrophage-targeting. Confocal imaging data showed that B/H@NPs@CpG-αmp displayed remarkably stronger fluorescent intensity in M2-like macrophages than the B/H@NPs@CpG complexes that lack of targeting ligands (Figure 2A). The sample identified as the M2p-blocked sample was pre-treated by pre-exposing it to high concentrations of M2pep peptide. The αmp-blocked sample was pre-treated by pre-exposure to high concentrations of α-peptide. Both samples displayed weak fluorescent intensity in pre-conditioned, M2-like macrophages (Figure 2A). Flow cytometry data showed the strikingly higher affinity of B/H@NPs@CpG-αmp to M2-like macrophages compared with B/H@NPs@CpG complexes (Figure 2B-C). In addition, we performed *in vitro* competition experiments to test the uptake of B/H@NPs@CpG-αmp nano-complexes by M1-like macrophages and M2-like macrophages. For the competition assay, M1-like and M2-like macrophages were seeded in 12-well plates in a 1:1 ratio. Then, B/H@NPs@CpG-αmp labeled with Cy5 was co-incubated with the mixed macrophages for 12 h. Next, the amounts of nano-complexes taken up by M1-like and M2-like were detected by flow cytometry. As shown in Figure S4A, M2-like macrophages take up much more B/H@NPs@CpG-αmp than M1-like macrophages. The same results are also observed in confocal images (Figure S4B -C). The B/H@NPs@CpG-αmp is effectively taken up by both M1-like and M2-like macrophages, while M2-like macrophages show a higher uptake rate due to their higher expression of surface receptors for M2pep and α-pep. All these data fully confirm that the nano-complexes of B/H@NPs@CpG-αmp with the double peptide targets -M2pep and α-peptide- demonstrate effective targeting capacity to M2-like macrophages *in vitro*.

After being taken up by macrophages, the cytotoxicity of the different formulations of the nano-complexes containing PBS, Hgp, H@NPs, B/H@NPs, B/H@NPs@CpG and B/H@NPs@CpG-αmp were evaluated using a proliferation/toxicity assay that quantified active dehydrogenases in macrophage cytoplasm (CCK-8 kit). As shown in Figure S2B, we observed negligible cytotoxicity of formulations toward macrophages in 24 h. We attribute this to the mild synthesis conditions and the use of biocompatible materials in formulations of the delivery system, as well as to the safety of baicalin as an immune-stimulant of macrophages.

### Anti-tumor properties of various nano-complexes *in vitro*

**Figure 3:**
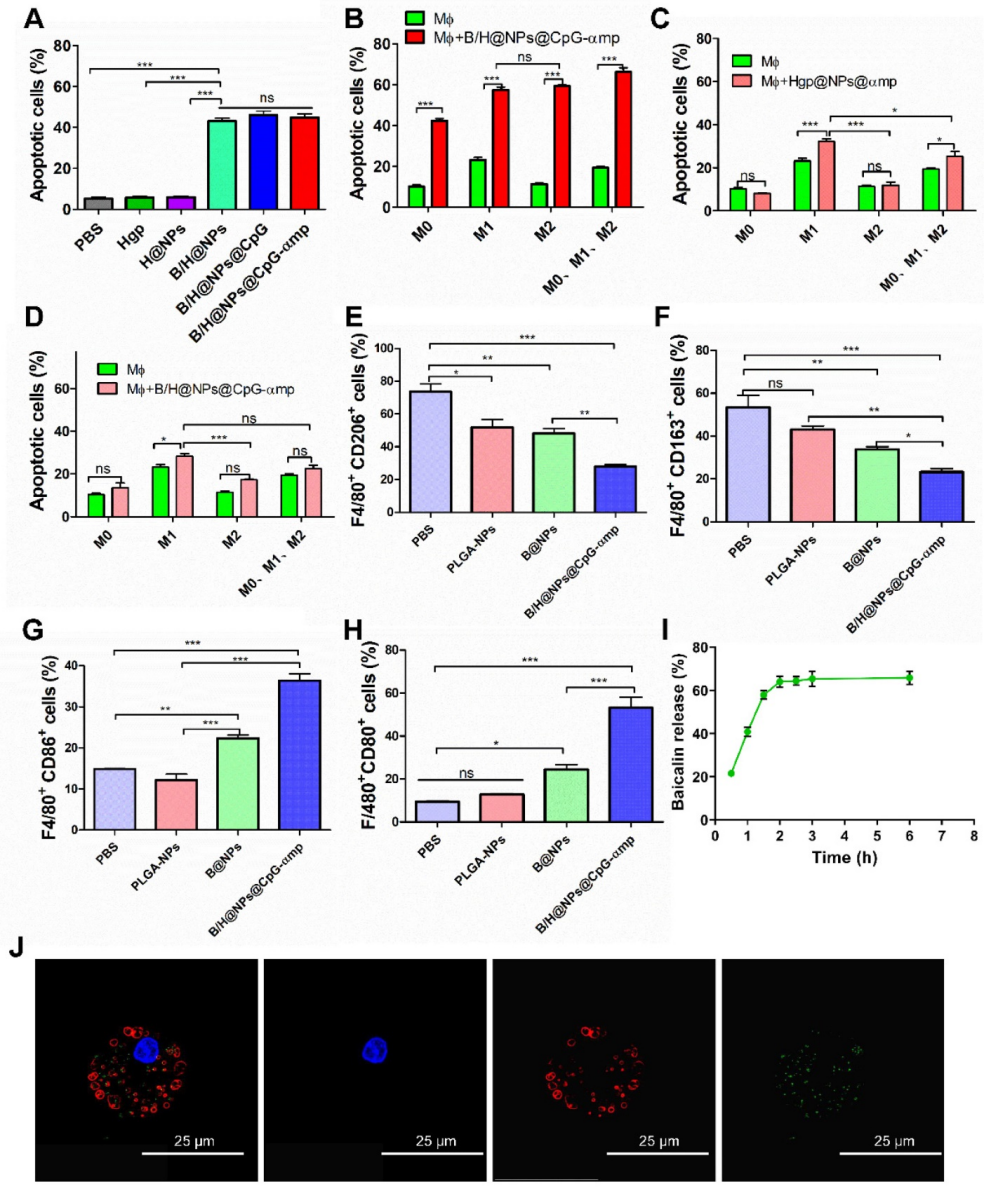
Capability of different nano-complexes to reverse TAMs phenotypes and anti-tumor mechanism *in vitro*. (A) Apoptosis analysis of B16 cells by flow cytometry using Annexin V-FITC/PI labeling at 24 h after treatment with different nano-complexes. (B) The Annexin V-FITC/PI apoptosis detection analysis of B16 cells co-cultured across Trans well plates with different types of macrophages and incubation with M2-like macrophages-targeting nano-complex: B/H@NPs@CpG-αmp. There are 2 × 10^4^/well T cells in this microenvironment. Data obtained at 24 h after treatment by flow cytometry. (C) The Annexin V-FITC/PI apoptosis detection analysis of B16 cells co-cultured with different types' macrophages and incubation with Hgp@NPs@αmp without baicalin and CpG. There are 2 × 10^4^/well T cells in this microenvironment. (D) The Annexin V-FITC/PI apoptosis detection analysis of B16 cells co-cultured across Trans well plates with different macrophages after incubating with B/H@NPs@CpG-αmp. There are no T cells in this microenvironment. (E, F) CD206 and CD163 expression in M2-like macrophages after treatment with different formulations. (G, H) The expression of CD86 and CD80 in M1-like macrophages after treatment with different formulations. Data obtained at 24 h after treatment by flow cytometry. (I) Images of the lysosomal escape of nano-complexes within macrophages; Blue: nucleus, Red: lysosomal fraction, Green: nano-complex. (J) The release percentages of baicalin from nano-complexes taken up by macrophages. Three independent experiments were analyzed in every group, n = 3. Data are expressed as the mean ± standard error of the mean (SEM). Differences between two groups were tested using an unpaired, two-tailed, Student's t-test. Differences among multiple groups were tested with one-way ANOVA followed by Tukey's multiple comparison. Significant differences between groups were expressed as follows: *P < 0.05, **P < 0.01, or ***P < 0.001.

Encouraged by the excellent uptake of nano-complexes in M2-like macrophages and the validation of the low toxicity, we next investigated the capacity of nano-complexes for killing B16 cells. It has been reported that baicalin inhibits Hela cells, lymphocytic leukemia, lung cancer, ovarian cancer, and human colorectal cancer cells 14, 16, 23. In this study, the baicalin-loaded nano-complexes displayed significantly greater capacity for inducing B16 cells into apoptosis compared to those without baicalin (Figure 3A). Figure 3 demonstrates that baicalin-loaded nano-complexes promote efficiently killing of B16 cells. As shown in Figure 3A, B/H@NPs@CpG and B/H@NPs@CpG-αmp exhibit no greater cytotoxicity to B16 cells than B/H@NPs. It is proposed that only baicalin exerts an anti-tumor response in this case, and the immuno-stimulant CpG does not directly kill tumors. The loaded CpG thus only plays an indirect role in killing tumor cells by activating the immune cells.

Some studies report that CpG carries out an anti-tumor role through activating immune cells and reversing M2-like macrophage phenotypes in a tumor microenvironment (M2-like TAMs). The activated TAMs subsequently secrete additional cytokines and present antigen to T cells that induces the anti-tumor immune response 11, 24-26. In this study, we simulated the tumor microenvironment *in vitro* using Trans well plates with tumor cells, TAMs and T cells. As shown in the summarized results of Figure 3B, a higher rate of tumor cell apoptosis is observed in all macrophage groups stimulated with B/H@NPs@CpG-αmp compared to the unstimulated macrophages. The macrophage samples combined with nano-complexes induced the greatest fraction of apoptotic tumor cells (69.72%, Figure 3B), compared to the fraction achieved by direct cytotoxicity utilizing an equal dose of the B/H@NPs@CpG-αmp nano-complexes in the absence of macrophages (42.76%, Figure 3A). These results suggest that baicalin & CpG-loaded nano-complexes that target M2-like TAMs-realize an additional, indirect anti-tumor toxicity through macrophage action. This indirect anti-tumor effect is induced through mediation by baicalin and CpG. We find that in the groups without B/H@NPs@CpG-αmp stimulation, macrophages with different phenotypes promote various levels of apoptosis (Figure 3B, green columns). M1-like macrophages show the greatest anti-tumor effect with 25.3% cells undergoing apoptosis, followed by the M0-like and M2-like macrophages with 10.4% and 11.6%, respectively. These results indicate that the macrophages with M0-like and M2-like phenotypes show poor ability to promote B16 tumor cell apoptosis. However, when macrophages with M0-like, M1-like, and M2-like phenotypes are co-incubated with B/H@NPs@CpG-αmp and T cells, their anti-tumor efficiency is increased greatly (Figure 3B, red columns). We also observed that there is no significant difference in promoting anti-tumor cytotoxicity between M2-like and M1-like groups after activation by the nano-complexes (Figure 3B, red columns). We hypothesize that the M2-like phenotype successfully reverts to anti-tumor macrophages by stimulation with the B/H@NPs@CpG-αmp nano-complexes. As the control experiment, we compared groups of macrophages (M0 *vs* M1 *vs* M2 *vs* phenotype mix) treated with nanoparticles without baicalin and CpG and evaluated tumor cell apoptosis. The results are shown in Figure 3C, where tumor cell apoptosis is observed to be significantly elevated for M1-like macrophages and the mixed group treated with nanoparticles without baicalin and CpG (specifically Hgp@NPs@αmp). In contrast, no difference was observed in M0 and M2-like macrophages. This result suggests that M1-like macrophages actively take up some antigen-carrying nanoparticles, and process and present antigen peptide to T cells. We presume that the activated T cells perform tumor cell killing. However, M2-like macrophages do not perform effective antigen delivery that induces anti-tumor immunity of T cells. We measured apoptosis in B16 cells that were co-cultured with different types of macrophages and incubated with B/H@NPs@CpG-αmp nano-complexes. These measurements show there are no T cells in this microenvironment. We also monitored B16 cell apoptosis after co-culturing with different macrophages activated by B/H@NPs@CpG-αmp. The results are shown in Figure 3D, where a slight increase in tumor cells apoptosis in the M0, M2, and mixed groups treated with B/H@NPs@CpG-αmp is observed. However, no significant difference between them is visible. The M1-like macrophages that were incubated with nano-complexes slightly increased tumor cell apoptosis from 21.3% to 28.33%. This is 11.52% higher than seen for M2-like macrophages. We hypothesize that the activated M1-like macrophages secret IL-12, IL-6, and other cytokines resulting in tumor cell death. Furthermore, baicalin loaded in B/H@NPs@CpG-αmp may be delivered directly to tumor cells and produce apoptosis. Despite this, the macrophages do not produce a large incidence of tumor cell death absent the T cells. This indicates the importance of T cells in inducing the anti-tumor immune response.

In the above experiments, baicalin and CpG were important for the anti-tumor response. We next identified the phenotypes of macrophages that were incubated with the nano-complexes. As shown in Figure 3E-H, the expression of CD206 and CD163 (the marker of M2-like macrophages) decreased, while the CD86 and CD80 expression markers (the marker of M1-like macrophages) significantly increased after exposure to B/H@NPs@CpG-αmp. These results are consistent with the tumor cell apoptosis data, which suggests that the anti-tumor efficiency is associated with macrophages phenotype reversion. We note that the baicalin-loaded in nano-complexes also promoted tumor cells apoptosis. In addition, the data indicate that macrophages reversed from M2-like to M1-like and were further activated. They then presented their Hgp antigen to neighboring T cells for the anti-tumor response. As shown in Figure 3I and 3J, the macrophages that were incubated with nano-complexes were also the secondary delivery carrier of baicalin to the tumor. We traced the course that the B/H@NPs@CpG-αmp nano-complexes took in escaping from lysosomes of macrophages and finally releasing baicalin to the extracellular space. The amount of baicalin released was determined to be over 60% of the initial loaded dose at 3 h after administration (Figure 3I). The released baicalin was sufficient for killing tumor cells.

Some literature has shown that certain chemotherapy drugs (DOX and paclitaxel) can be delivered to tumors using macrophages as carriers. Chemotherapy-loaded macrophages showed promising anti-cancer efficacy in terms of tumor suppression, life span prolongation, and metastasis inhibition, with reduced toxicity 20, 21. In the present study, nano-complexes loaded with baicalin showed the same tumor killing effect. However, due to the immunomodulatory effect of baicalin, the nano-complexes would show better anti-tumor effects *in vitro* and *in vivo*. The mixed macrophage group produced the highest tumor cell apoptosis among all the groups (Figure 3B). This interesting phenomenon might have benefited by nano-complexes targeting the M2-like TAMs, which could activate M1-like and M2-like macrophages. Although the mixed phenotype group of macrophages contained fewer M1-like macrophages than those in the M1-like macrophage group, the M2-like macrophages targeted by the B/H@NPs@CpG-αmp were reprogrammed to present characteristics of M1-like macrophages, which led to greater populations of M1-like macrophages. The M1-like macrophages further activated T cells that resulted in greater inflammatory cytokine secretion. This turn of events eventually promoted the maturation and activation of M0-like and M1-like cells. Therefore, we speculate that the macrophages with mixed phenotypes demonstrate better anti-tumor responses than other groups. Our results show that the nano-complexes with dual peptide targets are effectively engulfed by M2-like macrophages *in vitro*, which further releases CpG that converts M2-like phenotypes to M1-like ones. The M1-like macrophages not only secrete certain anti-tumor cytokines, but also activate T cells to kill tumor cells. In summary, we demonstrated that nano-complexes with dual peptide targets have dual immunotherapeutic and cytotoxic effects on B16 cells.

### Nano-complexes with dual peptide ligands effectively target M2-like TAMs in melanoma tumors *in vivo*

**Figure 4:**
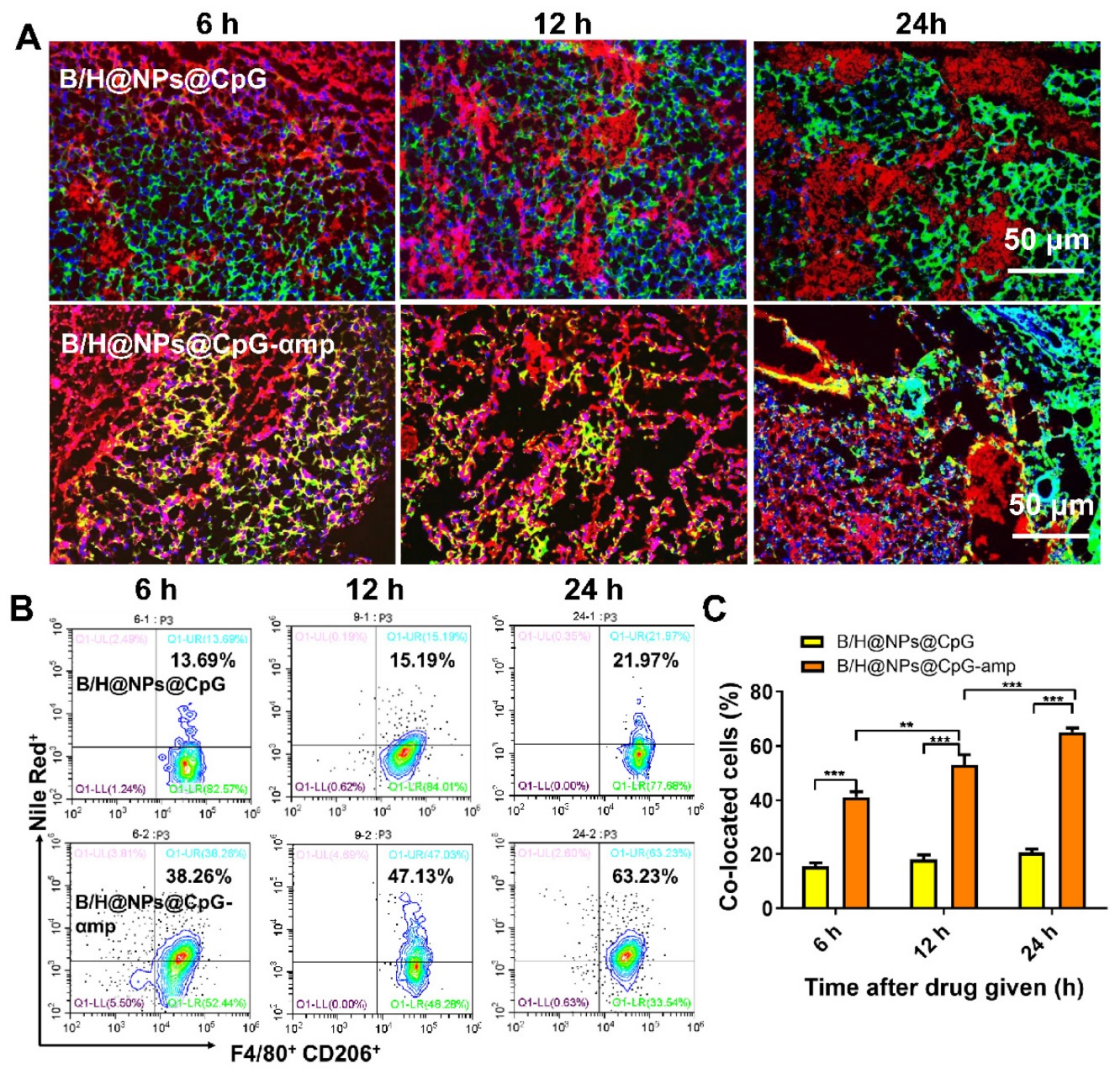
Evaluation of nano-complexes of B/H@NPs@CpG-αmp for *in vivo* targeting of M2-like TAMs. (A) Representative immunofluorescence images for detection of F4/80^+^ and CD206^+^ TAM targets using B/H@NPs@CpG and B/H@NPs@CpG-αmp nano-complexes at 6 h, 12 h, 24 h after treatment. Yellow color represents co-localization of nano-complexes and TAMs. Blue: cell nucleus, red: nano-complex, green: CD206^+^ TAMs. Scale bar: 50 µm. (B, C) Quantitative analysis of B/H@NPs@CpG and B/H@NPs@CpG-αmp targeting to F4/80^+^, CD206^+^ TAMs by flow cytometry. Three independent experiments were analyzed in every group, n = 3. Data are expressed as the mean ± standard error of the mean (SEM). Differences between two groups were tested using an unpaired, two-tailed Student's *t*-test. Differences among multiple groups were tested with one-way ANOVA followed by Tukey's multiple comparison. Significant differences between groups are expressed as follows: *P < 0.05, **P < 0.01, or ***P < 0.001.

When nano-complexes of B/H@NPs@CpG-αmp that target M2-like TAMs were delivered to M2-like macrophages, the M2-like phenotype was successfully reversed and activated *in vitro*. We next evaluated the targeting capability of B/H@NPs@CpG-αmp *in vivo*. To localize the nano-complexes, B/H@NPs@CpG and B/H@NPs@CpG-αmp, *in vivo,* they were labeled with fluorescent Nile-Red dye, and their localization at the tumor site was tracked. As expected, cryosections of tumor tissues showed that 6 h to 24 h after injection, B/H@NPs@CpG-αmp (red) nano-complexes co-localized with CD206^+^ TAMs (green) (co-location: yellow area, Figure 4A and Figure S5). This observation suggests that B/H@NPs@CpG-αmp effectively targets M2-like TAMs. We visually observed that the co-localization area (yellow) in the B/H@NPs@CpG-αmp group was conspicuously larger than co-localization in the B/H@NPs@CpG group. This result was further confirmed by flow cytometry analysis of cell suspensions from harvested tumor tissues. The nano-complexes targeting blank and dual M2-like cellular receptors was compared in tumor tissues* in vivo* at 6 h, 12 h and 24 h after intravenous injection using flow cytometry (Figure 4B, C). The M2-like TAMs were identified by labeling F4/80 and CD206 markers according to previous reports 6, 27. The flow cytometry results showed that F4/80^+^ CD206^+^ M2-like TAMs captured significantly more B/H@NPs@CpG-αmp compared with B/H@NPs@CpG complexes (Figure 4B). The co-localized cells in the treatment group of B/H@NPs@CpG-αmp were 3-fold more numerous than those in the treatment group of B/H@NPs@CpG (Figure 4C).

We also tracked the* in vivo* dynamic distributions of the targeting and non-targeting nano-complexes after intravenous administration. In this study, the melanoma tumors were first modeled by subcutaneous injection of melanoma (B16) cells into the right armpit of mice. After two weeks, the mice were intravenously given 100 μL Cy5-labeled formulations including Free CpG, B/H@NPs@CpG (non-targeting), B/H@NPs@CpG-mp (single peptide-targeting) and B/H@NPs@CpG-αmp (dual peptide-targeting). Then, the dynamic distributions of different formulations in various organs were monitored using an *in vivo* imaging system (KODAK, Carestream FX PRO) and flow cytometry. As shown in Figure S6, the free CpG is widely distributed and concentrates in the abdominal cavity, hardly reaching to the tumor site.

Before 24 h, most of the non-targeting formulations, such as the B/H@NPs@CpG nano-complex mainly distributes in liver, spleen and lungs, with only a small proportion reaching the tumor site, presumably due to the EPR effect. However, both single-targeted and double-targeted nano-complexes, such as B/H@NPs@CpG-mp and B/H@NPs@CpG-αmp, reach the tumor sites quickly, and further accumulate there gradually over time. This pattern is especially evident for the dual-targeted nano-complexes, such as B/H@NPs@CpG-αmp. After 72 h, a large amount of the dual-targeted nano-complexes accumulated at the tumor site, and little remained in liver (Figure R6 B). The dynamic distribution of the dual-targeting nano-complexes to various organs was also confirmed by flow cytometry data. These measurements show that the nano-complex accumulation at the tumor sites increased gradually over time (Figure S6C). Taken together, the nano-complexes with dual peptide targets, such as B/H@NPs@CpG-αmp, demonstrate superior M2-like TAMs-targeting capability *in vivo*. These observations pave the way for achieving specific delivery of therapeutic drugs (*e.g.*, CpG, baicalin and Hgp) to M2-like TAMs *in vivo*.

### Analysis of TAMs phenotype *in vivo* after intravenous administration of nano-complexes

**Figure 5:**
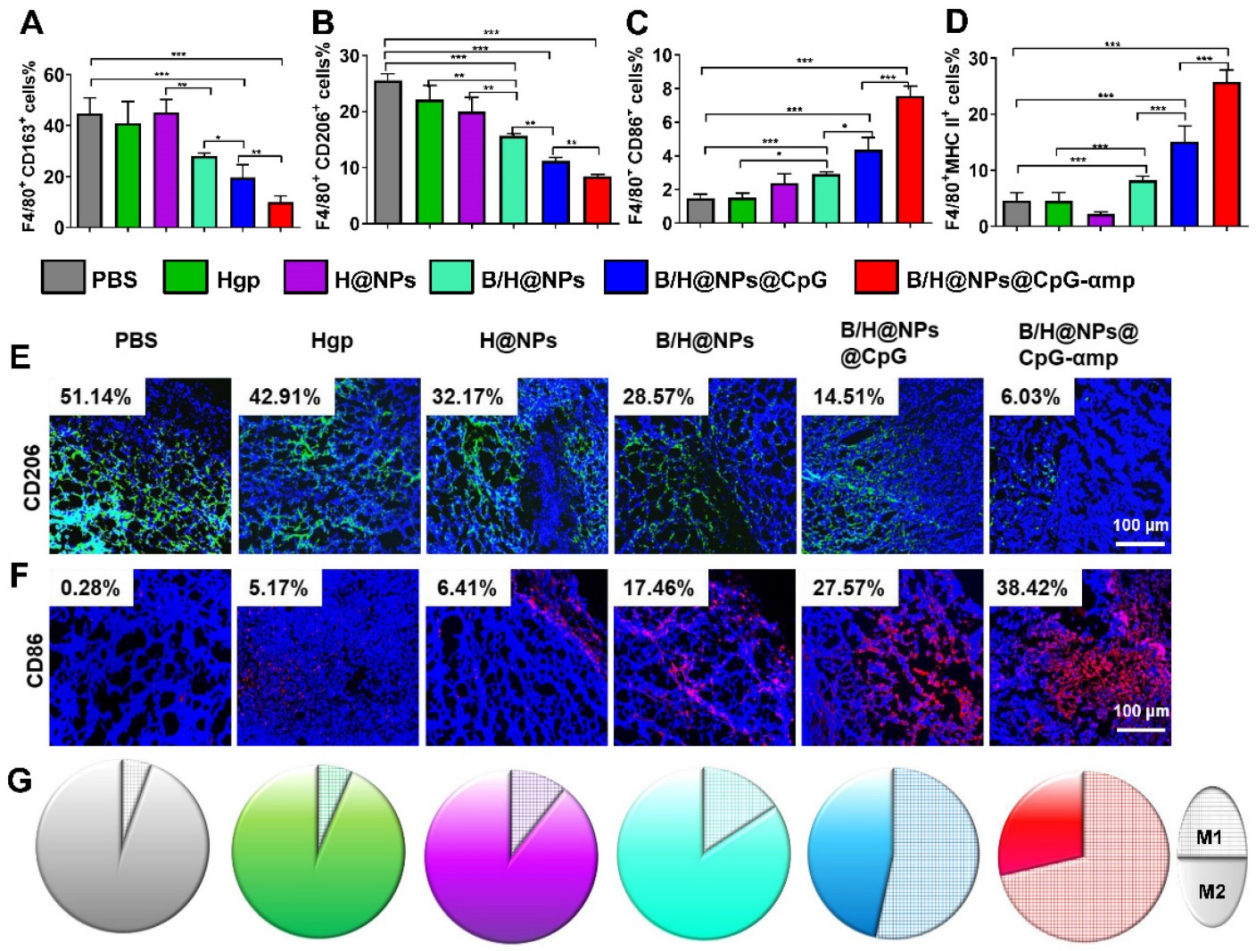
The TAMs phenotype reversion at tumor sites after treatment by different nano-complexes. The M2-like TAMs surface markers CD163 (A) and CD206 markers (B) together with M1-like surface markers CD86 (C) and MHC II markers (D) of TAMs were analyzed by flow cytometry. (E, F) The TAMs of M2-like (CD206) and M1-like (CD86) phenotypes were detected by immunofluorescence. Blue: cell nucleus, red: CD86^+^ cells, green: CD206^+^ cells. (G) The TAM phenotypes of M1-like (single color fill) and M2-like (hatched grid fill) area fractions within tumor tissue as represented respectively by CD86 and CD206 biomarkers after treatment with different nano-complexes. The data were analyzed by automatic multispectral imaging system (PerkinElmer Vectra II). For Figures E-G, the quantifications are shown in Figure S7A-C. Scale bar: 100 μm. Three mice were analyzed in every group (n = 3), and one representative image is displayed per group. Data are expressed as the mean ± standard error of the mean (SEM). Differences between two groups were tested using an unpaired, two-tailed Student's *t*-test. Differences among multiple groups were tested with one-way ANOVA followed by Tukey's multiple comparison. Significant differences between groups are expressed as follows: *P < 0.05, **P < 0.01, or ***P < 0.001.

The preceding results show that B/H@NPs@CpG-αmp nano-complexes effectively convert M2-like phenotypes into M1-like phenotypes* in vitro* and target M2-like TAMs *in vivo*. Here, the polarization and activation of TAMs in melanoma-bearing mice was examined using flow cytometry and immunofluorescence staining. The transformation of TAM surface co-stimulatory molecules reflects the phenotype reversion. For example, M1-like TAMs are associated with MHC II and CD86 surface markers, and M2-like TAMs with CD163 and CD206 markers. As shown in Figure 5A-B, CD163 and CD206 expression is suppressed by contact with the above nano-complexes, especially in the groups identified as B/H@NPs, B/H@NPs@CpG, and B/H@NPs@CpG-αmp nano-complexes. Expression of CD86 and MHC II proteins were up-regulated after administrating other baicalin-based nano-complexes (Figure 5C-D). These data indicate that the TAMs phenotype is most successfully reversed by the nano-complexes loaded with baicalin and CpG. These results suggest that baicalin and CpG play an important role in TAMs phenotype reversion, and explicitly show that the nano-complexes were successful at reprogramming from M2-like to M1-like TAMs at the tumor site. Furthermore, as shown in the immunofluorescence staining images, the nano-complex loading with baicalin and CpG in B/H@NPs, B/H@NPs@CpG, and B/H@NPs@CpG-αmp also significantly reduced the level of the CD206 surface marker observed in M2-like TAMs (in Figure 5E and Figure S7A, from 51.14% to 28.57%, 14.51%, and 6.03%, respectively, for B/H@NPs, B/H@NPs@CpG and B/H@NPs@CpG-αmp). By contrast, exposure to these nano-complexes greatly increased the M1-like TAM CD86 surface marker at the tumor location (in Figure 5F and Figure S7B, from 0.28%, to 17.46%, 27.57%, and 38.42% for B/H@NPs, B/H@NPs@CpG, and B/H@NPs@CpG-αmp, respectively). The B/H@NPs@CpG-αmp complexes were the most efficient to reverse M2-like to M1-like macrophages due to their effective targeting of M2-like TAMs. The greater the amount of B/H@NPs@CpG-αmps taken up by M2-like TAMs, the higher the CpG content that is released inside the cells. This further leads to the largest M2-like reversion and the highest T cell activation.

Figure 5G shows the area ratios for cells expressing the M1-like biomarker with respect to areas that display the M2-like biomarker (represented by CD86^+^ cells with the hatch-filled grid, and CD206^+^ cells with the solid filled color). These results indicate that the ratio of M1-like to M2-like TAM phenotypes within tumor tissue is conspicuously enhanced after exposure to the nano-complexes (Figure S7C)*.* B/H@NPs produce a slight increase of M1-like TAMs, and a small decrease of M2-like TAMs compared with the PBS, Hgp, and H@NPs control groups. baicalin & CpG-loaded nanoparticles surpass all three control groups in altering M1-like to M2-like area ratios (from 1:17.3 to1:5, from 1:14.5 to 1:5 and from 1:8.5 to 1:5 for PBS, Hgp, and H@NPs, respectively). This result suggests that baicalin and CpG-loaded nanoparticles also play a role in reversing M2-like to M1-like conversion of TAMs. It is surprising that the B/H@NPs@CpG-αmp nano-complexes promote greater phenotype conversion from M2-like to M1-like compared with B/H@NPs@CpG (from 1:0.4 to 1:0.87). Initially, the nano-complexes with dual peptide targets, such as B/H@NPs@CpG-αmp, are engulfed predominantly inside M2-like TAMs. Subsequently, the B/H@NPs@CpG-αmp releases most of its CpG into the target cells, which then carry out M2-like to M1-like reversion.

### Cytokines and infiltration of immune cells in the tumor microenvironment after treatment with nano-complexes

**Figure 6:**
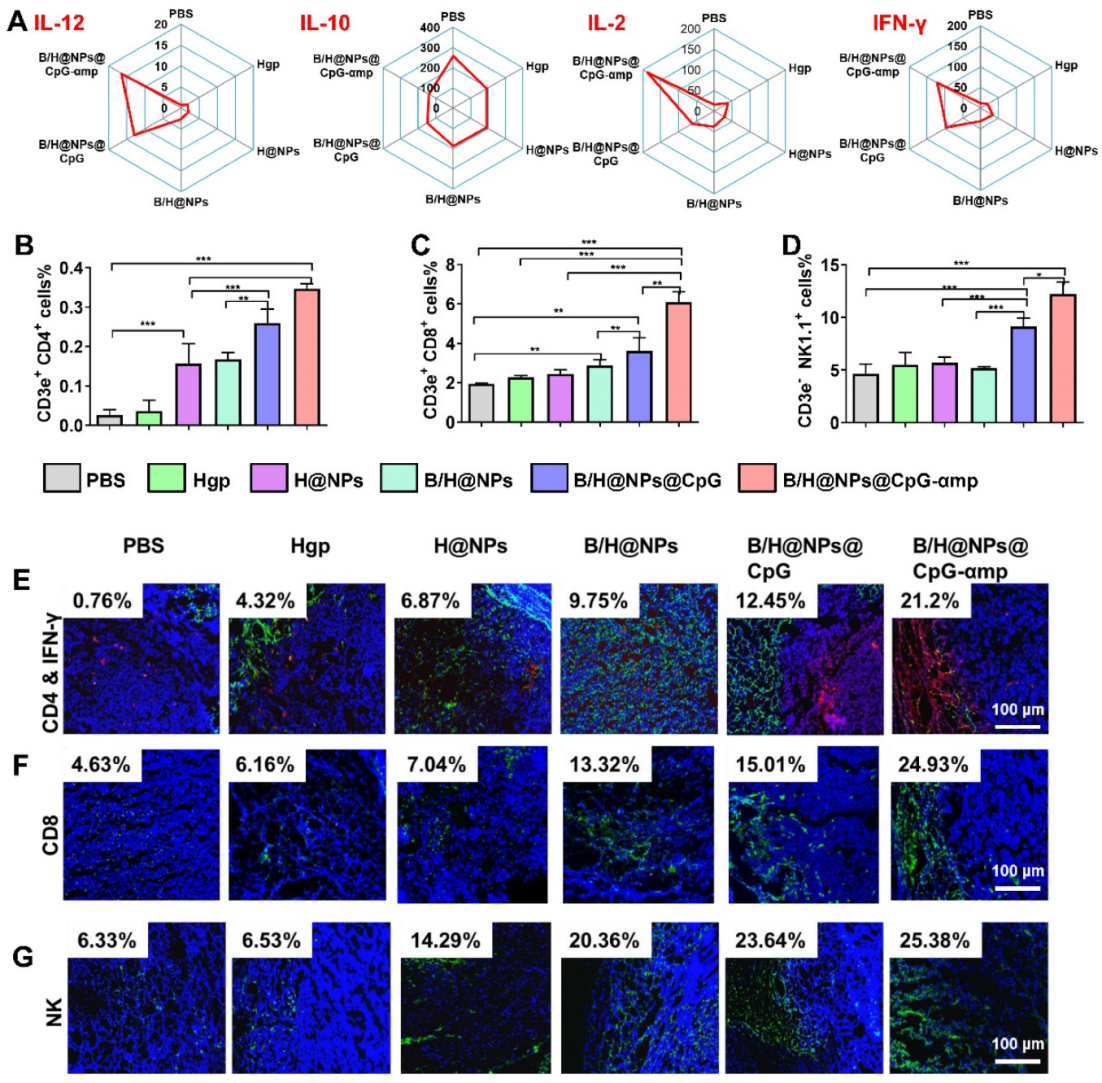
Different nano-complexes remodel the tumor microenvironment in B16 tumor-bearing mice. (A) The expression of IL-12, IL-2, IFN-γ and IL-10 cytokines at the tumor site were analyzed using an ELISA kit. (B, C, D) The activation of Th1 cells (CD4^+^ T), cytotoxic T cells (CD8^+^ T), and natural killer cells (NK) in tumors were examined via flow cytometry. (E, F, G) The infiltration of Th1 (CD4^+^/IFN-γ), CD8^+^, and NK cells at tumor sites examined by immunofluorescent staining. Blue: nucleus, Red: CD4^+^ cells, Green: IFN-γ, CD8^+^ cells, and NK cells, Yellow: the co-localization of CD4^+^ cells and IFN-γ. The data were analyzed by automatic multispectral imaging system (PerkinElmer Vectra II). Scale bar:100 μm. For E-G, the quantifications are shown in Figure S9A-C. Three mice were analyzed in every group (n = 3), and one representative image per group are displayed. Data are the mean ± SEM and representative of three independent experiments. Differences between two groups were tested using an unpaired, two-tailed Student's *t*-test. Differences among multiple groups were tested with one-way ANOVA followed by Tukey's multiple comparison. Significant differences between groups are expressed as follows: *P < 0.05, **P < 0.01, or ***P < 0.001.

Based on TAM phenotype reversion, cytokine expression levels are also important for evaluating the tumor microenvironment 28, 29. In this study, the supernatant from tumor tissues was collected for analysis of cytokines levels of IL-12, IL-10, IL-2, and IFN-γ. IL-12 promotes M1-like TAMs polarization and Th1 activation. IL-2 and IFN-γ are important for tumor immunotherapy 9, 30. IL-10 is a potent suppressive cytokine that produces angiogenesis and promotes M2-like TAM polarization that maintains the immune-tolerant microenvironment 30, 31. As shown in Figure 6A and S8A, the M1-like specific cytokine IL-12 significantly increases after treatment with the nano-complexes, especially after injections of the nano-complexes B/H@NPs@CpG and B/H@NPs@CpG-αmp. This suggests successful phenotype reversion of TAMs. IL-10 secretion from M2-like TAMs was greatly diminished after administration of the nano-complexes (from 269.3 ng/mL to 138.1 ng/mL), as shown in Figure 6A. Of all complexes tested, the groups treated with the B/H@NPs@CpG-αmp targeting M2-like TAMs showed the most evident up-regulation of IL-12 and down-regulation of IL-10. This strongest stimulation response is abetted by the powerful targeting capability and subsequently the highest uptake of the B/H@NPs@CpG-αmp by M2-like TAMs. The cytokine levels in this case further confirmed the greatest TAMs phenotype reversion by B/H@NPs@CpG-αmp.

The important cytokines for TAMs polarization and T cell activation were IL-2 and IFN-γ 10, 32. As shown in Figure 6A and Figure S8A, IL-2 was up-regulated by B/H@NPs@CpG-αmp (from 15.4 ng/mL to 181.7 ng/mL), and IFN-γ markedly increased from 11.6 ng/mL to 121.5 ng/mL after treatment. Some of the IFN-γ expression might be sourced from activated T cells in the tumor microenvironment. IL-12, IL-2, and IFN-γ played an important role in tumor immunity, which not only promoted M1-like TAMs polarization, but also stimulated the infiltration and activation of T cells and NK cells to tumor sites 27, 32, 33. The up-regulation of these pro-inflammatory cytokines signifies reconstruction of the tumor microenvironment, which is more favorable for suppressing tumors. These results demonstrate that the tumor microenvironment is most effectively benefited by effective B/H@NPs-CpG-αmp targeting to M2-like TAMs.

After validation of TAMs phenotype reversal and inflammatory cytokine secretion within tumor tissues, we assayed for activation and infiltration of T cells and NK cells at tumor locations using flow cytometry and immunofluorescence staining. It has been reported that the M1-like TAMs are capable of presenting antigen to T cells to elicit anti-tumor immunity through release of cytotoxins 29, 34. In this study, we found that H@NPs showed weak ability to activate T cells (CD4^+^ T cells 6.02-fold and CD8^+^ T cells 1.3-fold over the PBS group), as shown in Figure 6B-C. This implies that the nano-complexes with specific melanoma antigen alone is insufficient to activate T cells. However, as shown in Figure 6B-C, Th1 (CD4^+^ T cells) and CTL (CD8^+^ T cells) lymphocytes within tumor tissues are significantly elevated by B/H@NPs@CpG and B/H@NPs@CpG-αmp. The above results suggest that the cellular events occur in the following order: TAMs phenotype reversion is first induced by CpG and baicalin. M1-like TAMs overcome the barrier of antigen presentation and further recruit and activate additional T cells. Nano-complexes of B/H@NPs@CpG-αmp targeting M2-like TAMs showed the greatest percentage of both CD4^+^ T cells and CD8^+^ T cells in the tumor microenvironment. The CD4^+^ T cell percentages increased from 6.02-fold to 13.3-fold (Figure 6B), and the CD8^+^ T cell percentages increased from 1.3-fold to 3.2-fold (Figure 6C). Additional CD4^+^ T cells and CD8^+^ T cells infiltrated at tumor tissues and further augmented tumor cells killing. NK cells increased from 2.84% to 14.28% (Figure 6D). As shown co-localization of immunofluorescence in the micrographs of Figure 6E-G, massive Th1, CTL, and NK cell infiltration was observed after treatment with nano-complexes of B/H@NPs-CpG-αmp that target M2-like TAMs. The largest numbers of Th1^+^, CTL and NK cells in frozen sections of the tumor were observed in the group treated with B/H@NPs@CpG-αmp (reaching 21.2% Th1, 24.93% CTL, and 25.38% NK, Figure S9A-C). These results are consistent with the TAM phenotype reversion, which proved important for transforming the immunosuppressive to the immune-active tumor microenvironment. The activation of infiltrated T cells was detected by analyzing IFN-γ expression in tumor tissue. As shown in Figure 6E, immunofluorescence sections yielded a significant increase in IFN-γ expression in the tumor tissues after nano-complexes treatment, which was consistent with measurements of IFN-γ expression in tumor tissue supernatant. Our results indicate that the invading CD4^+^ T, CD8^+^ T, and NK cells were indirectly activated by the B/H@NPs-CpG-αmp targeting M2-like TAMs.

The detailed mechanism we propose is that the B/H@NPs-CpG-αmp are taken up by M2-like TAMs. The payloads are then released, which reverses M2-like to M1-like phenotypes. M1-like TAMs become further activated, which initiates an inflammatory cascade that releases cytokines that attract CD4^+^ T, CD8^+^ T, and NK cells. In summary, the B/H@NPs@CpG-αmp induced two types of immune responses in the tumor microenvironment. One was a direct M2-like TAMs phenotype reversion to M1-like phenotype, followed by a M1-like TAM activation. The other was an adaptive T-cell response, maintaining a persistent Th1 and CTL surveillance response through immune cell invasion and cytokine secretion. These results highlight the capability of B/H@NPs@CpG-αmp to promote immune cell responses, which ultimately enhances anti-tumor activity in a tumor microenvironment.

### Barriers to tumor growth and tumor angiogenesis in tumor microenvironment removed by nano-complexes *in vivo*

**Figure 7:**
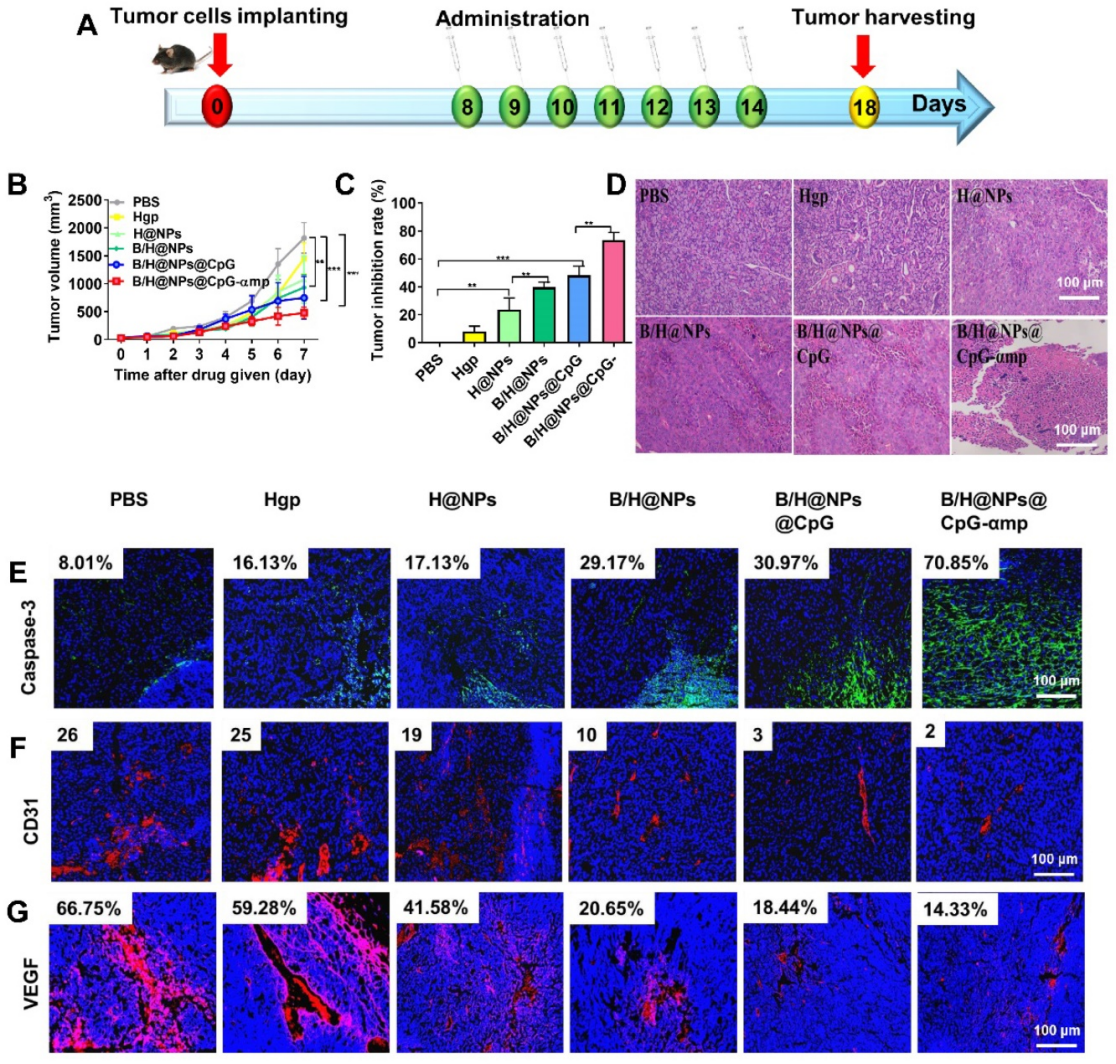
Effective anti-tumor and tumor microenvironment remodeling after treatment with different nano-complexes in the B16 tumor model. (A) Schematic illustration of the time sequence of administration of nano-complex to tumor-bearing mice. (B) Tumor volume from mice that received *iv infusion* containing different nano-complexes. (C) Tumor inhibition fractions after receiving *iv infusion* of various nano-complexes formulations. (D) Evidence of necrosis in tumors after treatment with different nano-complexes by hematoxylin and eosin (H&E) staining. (E) Caspase-3 analysis of tumor tissue indicating apoptotic cells by immunofluorescence in frozen tumor sections. (F) The number of vessels per image field is identified by CD31 label after treatment with different nano-complexes. (G) VEGF labeled by immunofluorescence indicates the quality of pro-angiogenesis secretion per image field after treatment with different nano-complexes. The data were analyzed by automatic multispectral imaging system (PerkinElmer Vectra II). Scale bar: 100 μm. For E-G, the quantifications are shown in Figure S11A-C. Three mice were analyzed in every group (n = 3), and one representative image per group is displayed. Data are the mean ± SEM and representative of three independent experiments. Differences between two groups were tested using an unpaired, two-tailed Student's *t*-test. Differences among multiple groups were tested with one-way ANOVA followed by Tukey's multiple comparison. Significant differences between groups are expressed as follows: *P < 0.05, **P < 0.01, or ***P < 0.001.

The aforementioned results prompted us to evaluate other therapeutic effects of the M2-like TAMs-targeting nano-complexes on B16 cell melanomas, a highly aggressive tumor model. The nano-complexes were injected 7 times at one day intervals starting 8 days after tumor cell implantation (Figure 7A). The tumor growth of the different groups was monitored daily after all treatments. Results showed that the nano-complexes of B/H@NPs, B/H@NPs@CpG, and B/H@NPs@CpG-αmp significantly inhibited tumor growth (Figure 7B). As summarized in Figure 7C, the tumor inhibition fraction of B/H@NPs increased from 23.2% to 40.7%, compared with the H@NPs control group. This result demonstrates that baicalin alone also produces a significant anti-tumor response. The incorporation of the immune-regulator CpG in B/H@NPs@CpG produced an enhanced anti-tumor response that is hypothesized to result from the combination of dual functions: One being the phenotype conversion of M2-like TAMs that produces direct cytotoxicity; the other being the capacity of M1-like TAMs to present antigen to T cells, which indirectly recruits the entire population of immune cells to respond through cytokine release. As expected, the B/H@NPs@CpG-αmp nano-complex exhibits the most intense anti-tumor effect, with the tumor inhibition fraction reaching 73.5%* in vivo*. The survival of the mice is shown in Figure S10B, and the groups receiving PBS, Hgp, and H@NPs died within 22 days. The groups receiving the baicalin/Hgp combination formulations saw remarkably prolonged survival times. Encouragingly, 83.3% of mice treated with B/H@NPs@CpG-αmp survived for more than 28 days. These survival studies further verify that B/H@NPs@CpG-αmp produces the most effective anti-tumor response.

Figure 7D shows extensive necrosis of tumor tissue in the HE sections from the groups treated with the nano-complexes of B/H@NPs@CpG-αmp. Eghbali-Feriz *et al.* reported that *Scutellaria barbata* extract (baicalin) inhibited the proliferation of hepatoma H22 cells through induction of cytochrome C, activation of caspase-3, procaspase 9, and the FNFR superfamily. In the present study, we determined the expression of caspase-3 in tumor tissues by immunofluorescence staining. The immunofluorescence images of Figure 7E and Figure S11A show that caspase-3 expression significantly increased in tumor tissues after treatment by the baicalin-loaded nano-complexes of B/H@NPs, B/H@NPs@CpG, and B/H@NPs@CpG-αmp. In contrast, the nano-complexes without baicalin, containing either PBS, Hgp, or H@NPs, did not provide evidence of significant caspase-3 expression. Our results suggest that the reverted TAMs exert a cytotoxic action and act as a second carrier whose further release of additional baicalin inhibits the proliferation of B16 cells. In addition to direct tumor killing by immune cells, our study also provides evidence of anti-angiogenesis as another important but indirect anti-tumor reaction. In the tumor microenvironment, M2-like TAMs are capable of stimulating tumor angiogenesis and tumor growth by secreting a wide variety of pro-angiogenic factors 35. After measuring the phenotype reversion of TAMs, we measured the expression of CD31 (angiogenesis) and VEGF (pro-angiogenic factors) from tumor tissues by immunofluorescence staining. As shown in Figure 7F and Figure S11B, CD31 staining demonstrates that tumors treated with B/H@NPs, B/H@NPs@CpG, and B/H@NPs@CpG-αmp show fewer blood vessels. Simultaneously, immunofluorescence confirmed less expression of VEGF (Figure 7G and Figure S11C) at tumor sites. It is well known that M2-like TAMs drive tumor progression and angiogenesis by expressing a wide range of growth and pro-angiogenic factors 9. From Figures 7F and 7G, we find that baicalin and CpG play an important role in inhibiting CD31 and VEGF expression due to their capacity for reversing TAM phenotypes. The M2-like TAM-targeting by nano-complexes of B/H@NPs@CpG-αmp show the best anti-angiogenesis effect at tumor sites. Therefore, we believe that the tumor retrogression induced by the nano-complexes was achieved because of TAM phenotype reversion, which further remodels the tumor microenvironment and promotes anti-tumor immunity.

The antitumor effect and immunomodulatory response of free baicalin, free CpG, and free baicalin and CpG (free B&C) were investigated in this study. The results (Figure S14) do not show TAMs phenotype reversion for free CpG or free baicalin and CpG. By contrast, the nano-complex groups, especially B/H@NPs@CpG-αmp, show significant reversion from M2-like to M1-like macrophages. To explain the action of NP formulations, free baicalin, CpG, and baicalin and CpG were added to tumor-bearing mice, as shown in Figure S12, and elaborated cytokines from tumor suspension cells were measured for these groups. Free baicalin and free B&C induce almost the same cytokine levels of IFN-γ and IL-12 as PBS, while free CpG induces significantly higher IFN-γ and IL-12 than PBS (Figure S12 A-B). However, the IFN-γ and IL-12 levels (23.89 pg/mL and 9.64 pg/mL) of the free CpG group were far less than the nano-complexes of B/H@NPs@CpG-αmp (143.89 pg/mL, 15.99 pg/mL) (Figure S12 A-B and Figure 6 A). Here, free baicalin, CpG and free B&C both induced significantly higher TNF-α levels than PBS. TNF-α is a tumor necrosis factor-related cytokine (Figure S12C). We speculate that TNF-α might be stimulated by baicalin chemotherapy and immune activation by CpG. As shown in Figure S12D-H, the tumor growth is inhibited by free baicalin, CpG, and baicalin and CpG. Tumor weight is also lighter under these chemotherapeutic agents than in PBS controls. However, the body weight of tumor-bearing mice treated with free baicalin, CpG, and free B&C showed a downward trend, which suggests the possibility of systemic toxicity by free baicalin, CpG, and baicalin and CpG (Figure S12F). In summary, the nano-complex formulations showed the advantages of less systemic toxicity and excellent tumor accumulation.

We performed additional experiments to determine the generality of anti-tumor responses and tumor micro-environmental remodeling effects from nano-complexes on other tumor-bearing mice (B16F10). The nano-complexes and other formulations were injected 7 times at one day intervals starting 8 days after B16F10 cell implantation (Figure S13 A). The tumor size was monitored daily after all treatments. Results show that free baicalin, free baicalin and CpG, B/H@NPs@CpG, and B/H@NPs@CpG-αmp significantly inhibit tumor growth, especially the group of nano-complexes such as B/H@NPs@CpG and B/H@NPs@CpG-αmp (Figure S13B-C). As summarized in Figure S13E, the tumor inhibition rate of free baicalin was over 60%. As expected, the B/H@NPs@CpG and B/H@NPs@CpG-αmp formulations exhibited much better anti-tumor effects, with tumor inhibition rates of 79.5% and 84.7% *in vivo*, respectively (Figure S13-E). These results further demonstrated the efficacy of the B/H@NPs@CpG and B/H@NPs@CpG-αmp nano-complexes on B16F10-bearing mice. In addition, the polarization of TAMs in the above animal model was also examined using flow cytometry. The transformation of TAM surface co-stimulatory molecules reflects their phenotype reversion. In this study, we employed multiple surface markers such as CD163 and CD206 to identify M2-like TAMs, and CD86 and CD80 to identify M1-like phenotypes to test their phenotype reversion. As shown in Figure S14A-B, expression of CD163 and CD206 was suppressed by the above nano-complexes, especially in the B/H@NPs@CpG and B/H@NPs@CpG-αmp groups. CD86 and CD80 expression was up-regulated upon administrating different nano-complexes (Figure S14C-D). However, there was no significant difference in TAMs surface markers for the groups of free baicalin, free CpG, and free baicalin and CpG, which indicates the successful TAMs phenotype reversion by B/H@NPs@CpG and B/H@NPs@CpG-αmp nano-complexes. Furthermore, these nano-complexes induced greater active immune cells infiltration into tumor sites, as shown in Figure S14D-E. The results show that the TAM targeting nano-complexes like B/H@NPs-CpG-αmp showed the highest percentage of both CD4^+^ T cells and CD8^+^ T cells invading the tumor. To summarize, these nano-complexes achieve anti-tumor effects by remodeling the tumor microenvironment, particularly by reversing the TAM phenotype and inducing immune cell infiltration. We speculate that free baicalin mostly kills tumors by chemotherapy and induces some CTL cell infiltration that exerts anti-tumor effects. Therefore, compared with free baicalin and free CpG, the nano-complexes achieve their anti-tumor effects by remodeling the tumor microenvironment, such as by reversing TAM phenotypes and inducing immune cell infiltration.

### Effects of the nano-complex on tumor metastasis

**Figure 8:**
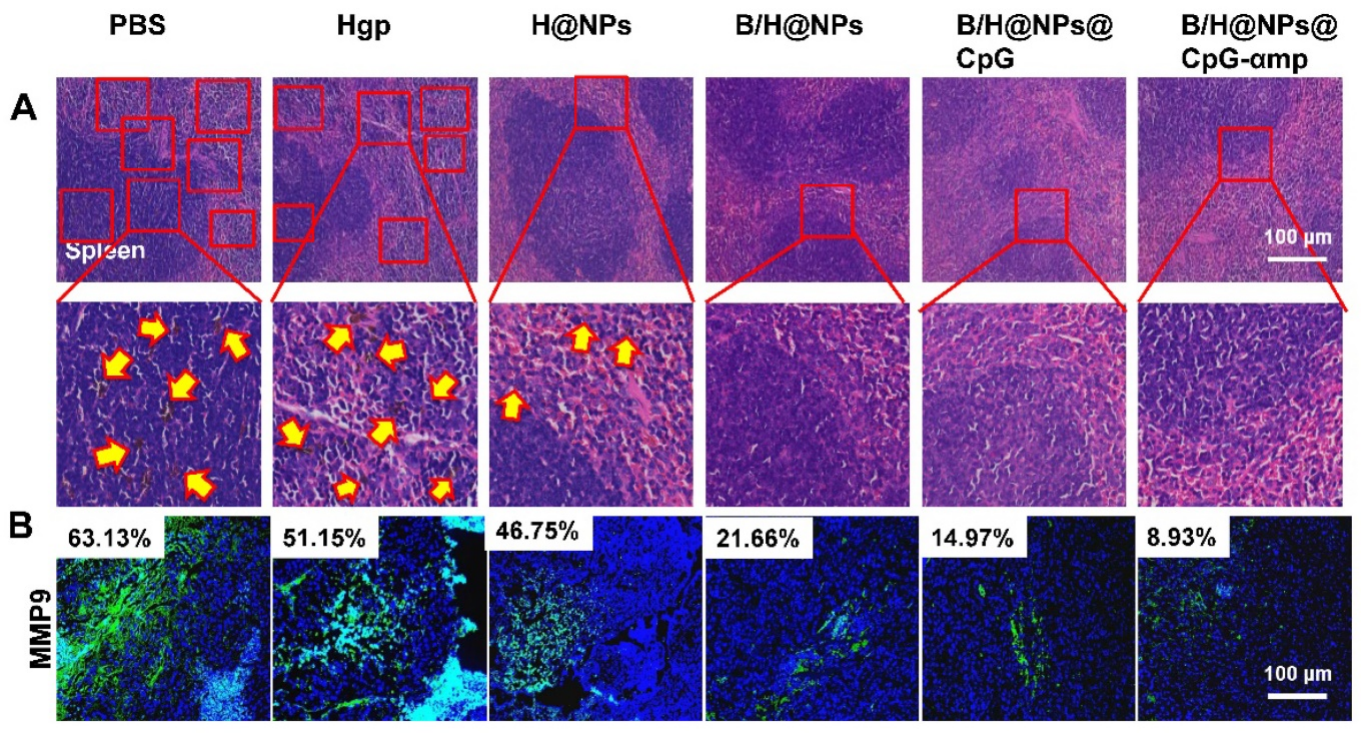
The different nano-complex formulations decreased metastasis of tumor cells from tumor sites. (A) Spleen metastasis indicated by the yellow arrow showing hematoxylin and eosin (H&E) staining. (B) Matrix metalloproteinases (MMPs) associated with metastasis were analyzed by immunofluorescence staining of MMP9. Blue: nucleus, Green: the expression of MMP9. The data were analyzed by automatic multispectral imaging system (PerkinElmer Vectra II). Scale bar, 100 μm. For B, the quantification is shown in Figure S16. Three mice were analyzed in every group (n = 3), and one representative image per group is displayed. Data are the mean ± SEM and representative of three independent experiments.

B16 melanoma is highly aggressive and straightforwardly metastatic. The capacity of nano-complexes to inhibit tumor metastasis was investigated. Metastatic melanoma cells were found in spleen in varying degrees for the PBS, Hgp, and H@NP treated animals (Figure 8A). We used melanoma antibody HMB-45 to mark the spleen tissue of the PBS group, which was retained from the previous experiment. From the results shown in Figure S15, the black colored cells transferred to the spleen were melanoma, which was consistent with H&E staining results. However, no sign of metastasis was observed in the mice treated with B/H@NPs, B/H@NPs@CpG, and B/H@NPs@CpG-αmp, as shown in Figure 8A. These results suggest that all nano-complexes are anti-metastatic to varying degrees, especially the nano-complexes of B/H@NPs@CpG-αmp that target M2-like TAMs. It has been reported that the expression of matrix metalloproteinase (MMP9) in cancer cells promotes tumor metastasis 23, 36. We examined MMP9 expression at tumor sites via immunofluorescence staining in the B16 model. As shown in Figure 8B and Figure S16, MMP9 decreases with baicalin-loaded nano-complexes. The greatest decrease was observed in the groups treated with the nano-complexes of B/H@NPs@CpG-αmp (fractional area of MMP9 involvement 8.93% compared to 63.13% in the PBS control group). Much literature has reported that TAM phenotype reversion reduces MMPs expression and tumor cells metastasis 9, 20, 37, 38. Our results are similar. Baicalin-loading nano-complexes, especially M2-targeting nano-complexes, exhibit an anti-metastatic effect through lowering MMP9 expression in the tumor microenvironment. At the same time, the nano-complexes increase expression of IL-12, IFN-γ, and IL-2 cytokines at tumor sites (Figure S8A).

Therefore, tumor microenvironment remodeling also inhibits metastases by the dual mode model (M2-like TAMs phenotype reversion and immune cells activation), which was shown to be active for the nano-complexes of B/H@NPs@CpG-αmp. These modes were found to be favorable for promoting pro-inflammatory cytokines secretion and anti-tumor immunity. Huang *et al.* reported that M2-like TAMs reversion greatly decreased VEGF and MMP9 expression in tumor tissues 9. In summary, the as-prepared B/H@NPs@CpG-αmp nanoplexes not only reduced the primary melanoma lesion, but also inhibited tumor cell metastasis by lowering VEGF and MMP9 expression in the tumor microenvironment.

### Priming of normal tissues with nano-complexes to retard tumor growth

**Figure 9:**
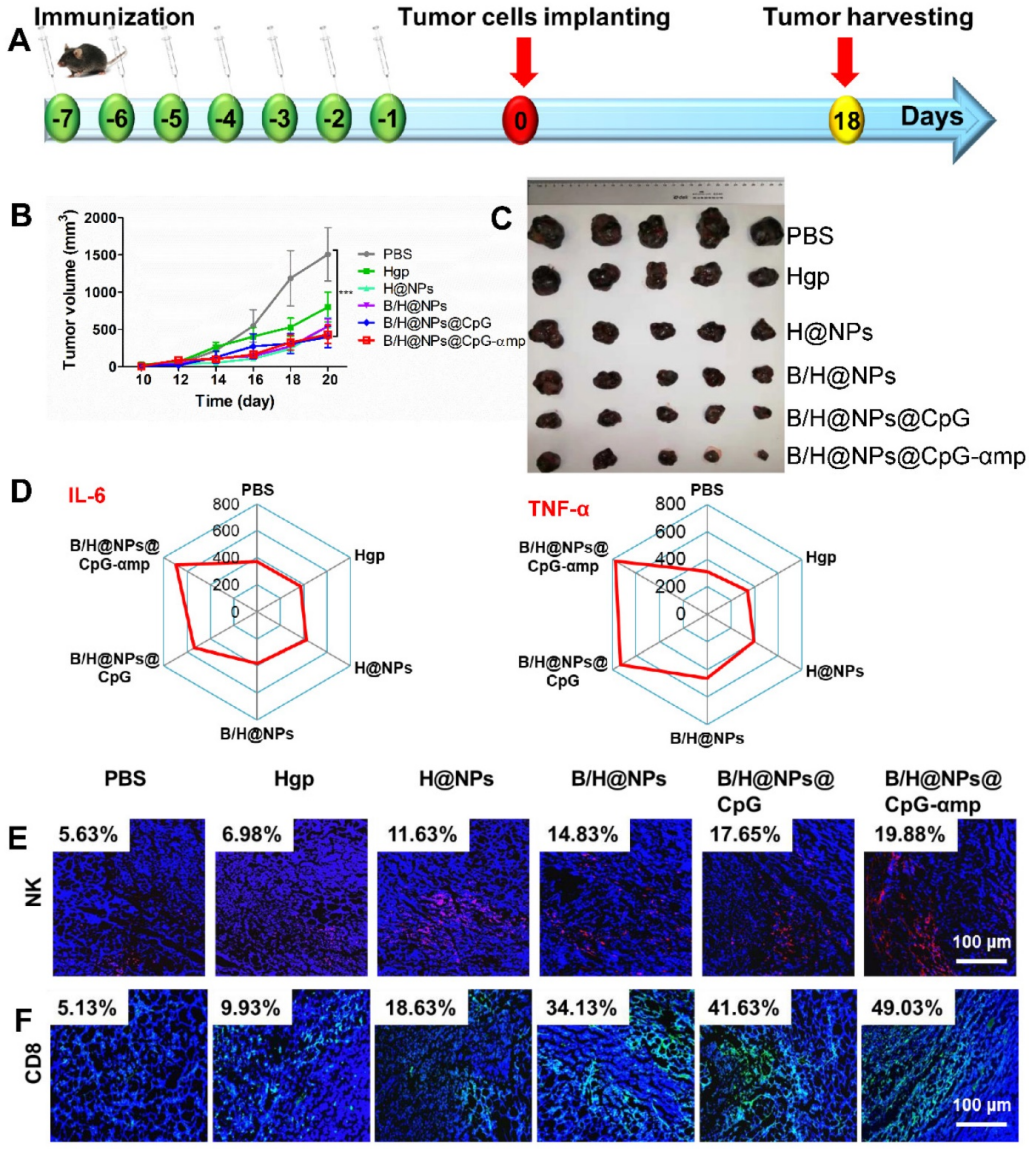
Tumor inhibition determined after receiving* i.v.* injections of nano-complexes as preventive vaccination utilizing leukocyte priming. (A) Schematic illustration of timing schedule for administration. (B) Tumor volume in animals that received *i.v.* injections of nano-complexes. (C) Images of tumors harvested from mice in each treatment group. (D) The expression of IL-6 and TNF-α in serum of tumor-bearing mice analyzed using an ELISA kit. For B-D, data are the mean ± SEM and representative of five independent experiments (n = 5). Significant differences between groups were expressed as follows: *P < 0.05, **P < 0.01, or ***P < 0.001. (E, F) The infiltration of CD8^+^ T cells and NK cells at tumor sites was examined by immunofluorescent staining. Blue: nucleus, Red: NK cells, Green: CD8^+^ T cells. The data were analyzed by automatic multispectral imaging system (PerkinElmer Vectra II). For E and F, the quantification is shown in Figure S18. Scale bar, 100 μm. Three mice were analyzed in every group (n = 3), and one representative image per group is displayed. Data are the mean ± SEM and representative of three independent experiments.

Based on the impressive anti-tumor properties of the nano-complexes *in vivo*, we investigated their effects as a preventative formulation. Six different nano-complexes were used to immunize normal C57 mice for 7 consecutive days (Figure 9A). On the day after the last vaccination, the mice were inoculated subcutaneously with 5 × 10^5^ B16 cells. Before inoculation, there was no significant difference in mice body weight among the six groups (Figure S17A), which suggests no systematic toxicity for any of these formulations. As shown in Figure 9B and Figure S17B, administration of the baicalin and CpG-loading nano-complexes significantly delayed tumor growth compared to the PBS group. Figure 9C shows the image of harvested tumor sections at 18 days after implanting the tumor cells.

The systemic anti-tumor cytokines of IL-6 and TNF-α were measured to assess immune priming at the systemic level. The serum cytokines of IL-6 and TNF-α were significantly up-regulated for the groups treated with different nano-complexes that were loaded with baicalin/CpG (Figure 9D and Figure S17C). As expected, these results were consistent with the results obtained from the tumor-bearing mice treatment by the same nano-complexes. The baicalin and CpG loaded nano-complexes of B/H@Ps, B/H@NPs@CpG, and B/H@NPs@CpG-αmp demonstrated better preventative resistance to tumor growth compared with other groups. The nano-complexes of B/H@NPs@CpG-αmp showed the best anti-tumor response. These results indicate that tumor suppression is possibly related to systemic activation of the immune system. We monitored the infiltration of NK cells and CD8^+^ T cells at the tumor location by means of immunofluorescent staining. As shown in Figure 9E-F and Figure S18A-B, the NK cells and CD8^+^ T cells within tumor tissue were significantly elevated after treatment with nano-complexes, especially for the groups exposed to B/H@NPs, B/H@NPs@CpG, and B/H@NPs@CpG-αmp payloads. The results suggest payloads of baicalin and CpG in nano-complexes assist Hgp to activate leukocytes and inhibit tumor progression.

The above results indicate that nano-complexes have the potential to act as an anti-tumor vaccine. There are many phagocytic immune cells *in vivo* such as DCs and macrophages, and these cells can actively ingest nano-complexes. The nano-complexes then release payloads such as antigen, baicalin, and CpG inside these cells. Subsequently, these cells are activated, process and present antigen that further induces activation of other immune cells such as CD4^+^ and CD8^+^ T cells. These activated immune cells then release cytokines that inhibit tumor growth. Therefore, when tumor cells are implanted in immunized mice, the systemic immune response retard tumor growth.

**Figure 10:**
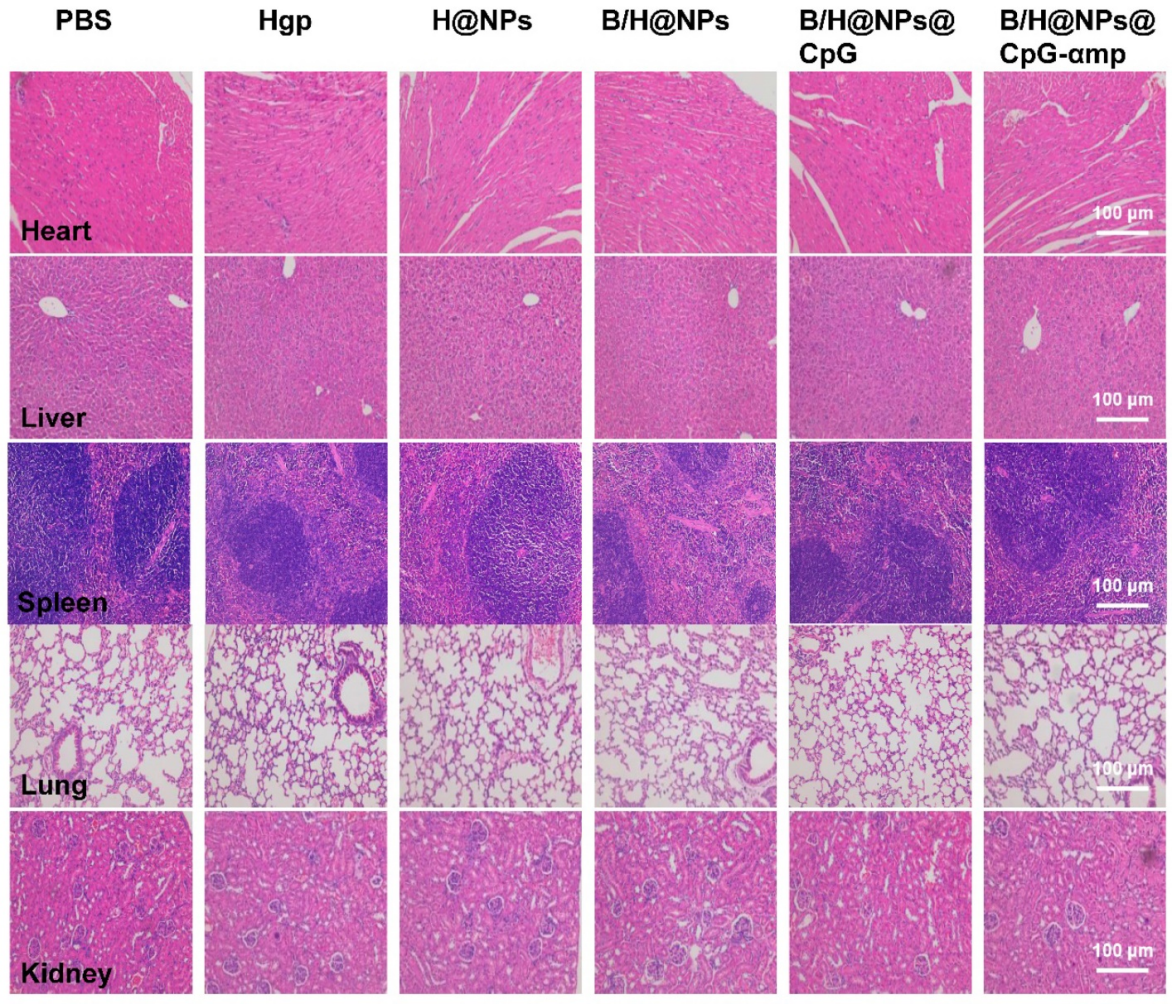
*In vivo* toxicity of nano-complex formulations. H&E-stained slice images of major organs from the different groups.

The *in vivo* toxicity for each of the nano-complex formulations was evaluated by immune histochemical analysis. As shown in Figure 10, few histological abnormalities were found in major organs of any of the groups receiving different nano-complexes. These results suggest that the combination nano-complexes, especially for the B/H@NPs@CpG-αmp nano-complex, exhibits almost no toxicity and offers satisfactory protection for tumor cell challenge. In addition, the toxicity *in vivo* of free baicalin, free CpG and free B&C (free baicalin and CpG) was evaluated by immune histochemical analysis. As shown in Figure S19, the mice treated with free baicalin and CpG showed no obvious damage to the heart, kidney, and lung tissues, compared with the PBS negative control. The alveoli in lung tissues were slightly dilated, however. In liver tissues, an inflammatory vascular response was observed after treatment with free baicalin and CpG. The blood vessels in the inflammatory foci expanded and became filled with red blood cells (white arrow) that was accompanied by inflammatory cell infiltration (orange arrow), especially in the CpG group. Free baicalin and CpG had significant effects on the morphology of spleen tissue, as shown in the figure S19. Compared with the PBS group, the red and white pulp regions of the spleen tissue in the experimental group became unclear, and many inflammatory cells had infiltrated. These results suggest that free baicalin and CpG might have some cytotoxicity on mice organs, which might also result in weight loss. The nano-complexes we formulated as carriers of free baicalin and CPG deliver these drugs to the tumor site in sufficient quantities to achieve an anti-tumor effect with targeting specificity that reduces systemic toxicity.

## Conclusion

A nano-complex that targets M2-like macrophages was successfully formulated for melanoma treatment. The nano-complex was shown to effectively reverse the TAM phenotype phase and further remodel the solid tumor microenvironment. PLGA nanoparticles were fabricated to encapsulate baicalin and/or Hgp antigen, and their surfaces were coated with polydopamine for ligand attachment. The nanoparticles were further decorated with M2pep and α-peptide ligands to form nano-complexes that target M2-like TAMs. The results of the *in vitro* and *in vivo* studies showed that the as-prepared nano-complexes successfully targeted and localized at M2-like TAMs and reversed their phenotypes to M1-like phenotypes. The activated M1-like TAMs acted as antigen presenting cells to present Hgp antigens to neighboring T cells for further activation of the inflammatory cascade to enhance the tumor killing function.

The reversion of M2-like TAMs to M1-like phenotype and activation of T cells significantly remodeled the tumor microenvironment in such manner that it resulted in immune cell recruitment and enhanced anti-tumor therapy. The substantial retardation of tumor growth was observed in tumor-bearing mice after intravenous administration of the nano-complexes for one week. Collectively, these results suggest that the TAM-targeting nano-complexes offer significant potential for treating and preventing melanoma by reversing M2-like TAMs and remodeling the tumor microenvironment.

## Supplementary Material

Supplementary figures and table.

## Abbreviations

APC: antigen present cells
B: baicalin
B cells: B lymphocyte cells
BMDMs: bone marrow-derived macrophages
CPG-ODN: cytosine-phosphate-guanine oligonucleotide
CD: co-stimulatory molecules
Con-A: concanavalin A
CCK-8: colorimetric cell counting Kit-8
CpG: cytosine-phosphate-guanine
CLSM: confocal laser scanning microscopy
CTL: cytotoxic T lymphocytes
DMEM: Dulbecco's modified Eagle's medium
DAPI: 4,6-diamino-2-phenyl indole
DLS: dynamic light scattering
DOX: doxorubicin
EE: encapsulation efficiency
ELISA: Enzyme-linked immunosorbent assay
FBS: fetal bovine serum
HPLC: high-pressure liquid chromatography
Hgp: tumor-associated antigen
HE: hematoxylin and eosin
IL: interleukin
IFN-γ: interferon-γ
LPS: lipopolysaccharide
LE: drug-loading efficiency
MFI: mean fluorescence intensity
M-CSF: macrophage colony stimulating factor
MHC: major histocompatibility complex
Mefp-5: mytilus edulis foot protein-5
MMP9: matrix metalloproteinase 9
M2pep: M2-like macrophage binding peptide
NPs: nanoparticles
NK: natural killing cells
OD: optical density
PLGA: poly(lactic-co-glycolic acid)
pD: polydopamine
PBS: phosphate-buffered saline
PDI: polydispersity index
R: tumor inhibition rate
RPMI: memorial institute 1640 medium
SEM: scanning electron microscopy
TEM: transmission electron microscopy
PI: propidium iodide
SR-B1: scavenger receptor B type 1
T: T lymphocyte cells
Th: T helper lymphocytes
TAMs: tumor-associated-macrophages
TNF-α: Tumor necrosis factor-α
V: relative tumor volumes
VEGF: vascular endothelial growth factor
W/O: water-in-oil
W/O/W: water-in-oil-in-water
