# Supplementary material for: Tumor microenvironment remodeling and tumor therapy based on M2-like tumor associated macrophage-targeting nano-complexes
Source: Theranostics. 2021 Jan 1;11(6):2892–916. doi: 10.7150/thno.50928 (PMC7806477; doi:10.7150/thno.50928)
Supplement: Supplementary file 1 — Supplementary figures and table. [file thnov11p2892s1.pdf]

Tumor microenvironment remodeling and tumor therapy based on M2-like tumor associated macrophage-targeting nano-complexes

Shulan Han[a,b], Wenjie Wang[a] , Shengfang Wang[a], Tingyuan Yang[b], Guifeng Zhang[b], Di Wang[c], Ruijun Ju[c], Yu Lu[d], Huimei Wang[a*], Lianyan Wang[b*]

[a] *College of Chemistry, Chemical Engineering and Resource Utilization, Northeast Forestry University. Harbin 150040, P. R. China*

[b] *Key Laboratory of Green Process and Engineering, State Key Laboratory of Biochemical Engineering, Institute of Process Engineering, Chinese Academy of Sciences, Beijing 100190, P.R. China*

[c] *Beijing Institute of Petrochemical Technology, Beijing 102617, P.R. China*

[d]*Institute of Veterinary Immunology &Engineering，Jiangsu Academy of Agricultural Sciences, Nanjing 210014, Jiangsu, P.R. China*

** Corresponding author.*

Address correspondence to: whm0709@163.com

wanglianyan@ipe.ac.cn (L.-Y. Wang)

Tel/Fax: 010-62574303

Supporting information

Table S1. The composition of different nanoparticles.

Name	Carrier	Content	Targeting Peptide
PBS	/	PBS	/
Hgp	/	Hgp100$_{25\text{-}33}$	/
H@NPs	PLGA-NPs	Hgp100$_{25\text{-}33}$	/
B/H@NPs	PLGA-NPs	Baicalin and Hgp100$_{25\text{-}33}$	/
B/H@NPs@CpG	PLGA-NPs	Baicalin, Hgp100$_{25\text{-}33}$ and CpG	/
B/H@NPs@CpG-mp	PLGA-NPs	Baicalin, Hgp100$_{25\text{-}33}$ and CpG	M2pep
B/H@NPs@CpG-αmp	PLGA-NPs	Baicalin, Hgp100$_{25\text{-}33}$ and CpG	α-pep and M2pep

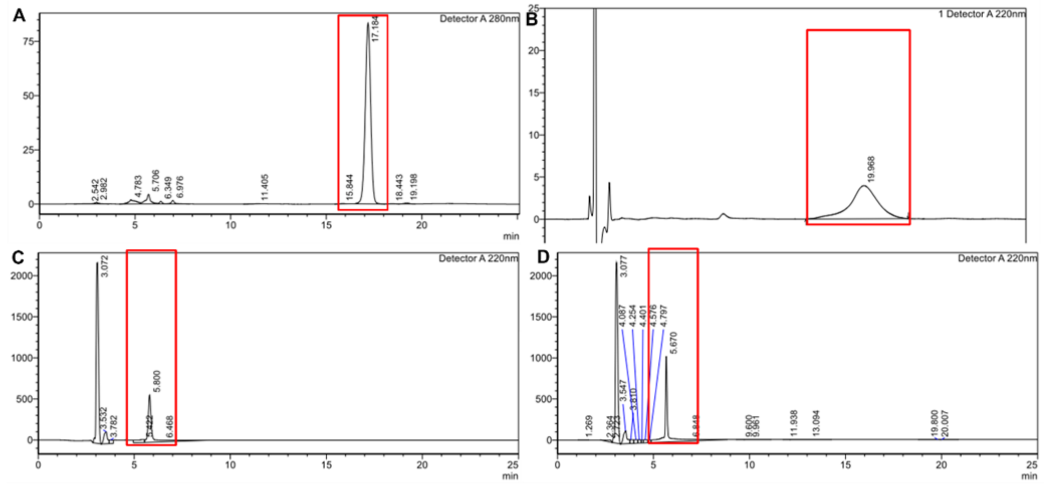

Figure S1. Drug loading of baicalin (A), Hgp100$_{25\text{-}33}$ antigenic peptide (B), α-peptide (C), and M2pep (D) in nano-complex detected by HPLC.

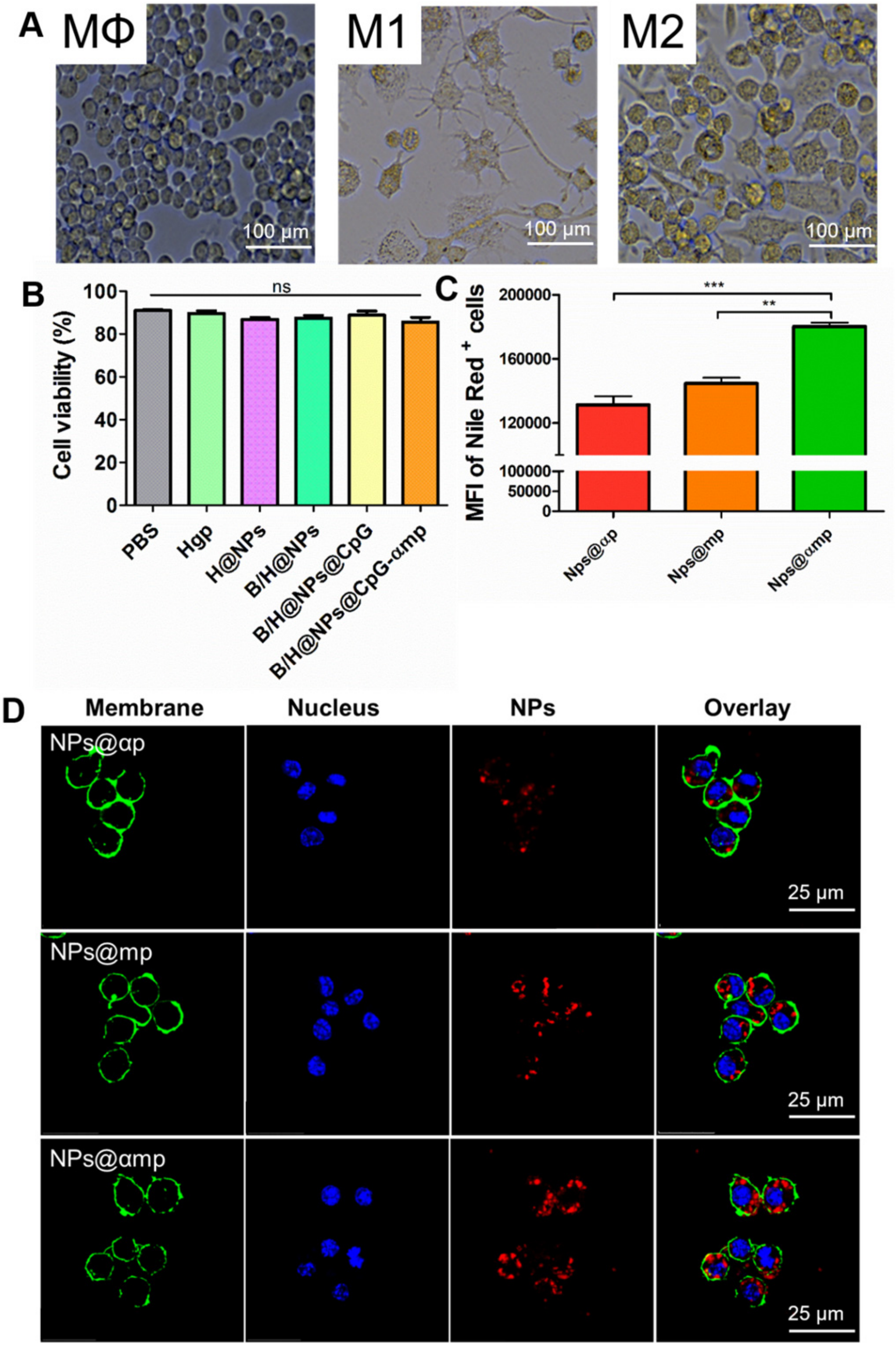

Figure S2. Analysis of the cytotoxicity and targeting ability of various nano-complex formulations in macrophages. *In vitro* culture and induced differentiation of MΦ, M1-like and M2-like

macrophages (A). Cytotoxicity of various nano-complexes against macrophages after treatment for 24 h (B). The M2-like macrophage-targeting capability of nano-complexes with different target peptides (NPs@αp, NPs@mp, and NPs@αmp) was analyzed using flow cytometry and confocal microscopy (C, D). The MFI is defined as the mean fluorescence intensity. Data are expressed as the mean ± standard error of the mean (SEM), n = 3. Differences between two groups were tested using an unpaired, two-tailed Student's t-test. Differences among multiple groups were tested with one-way ANOVA followed by Tukey's multiple comparison. Significant differences between groups are expressed as follows: *P < 0.05, **P < 0.01, or ***P < 0.001.

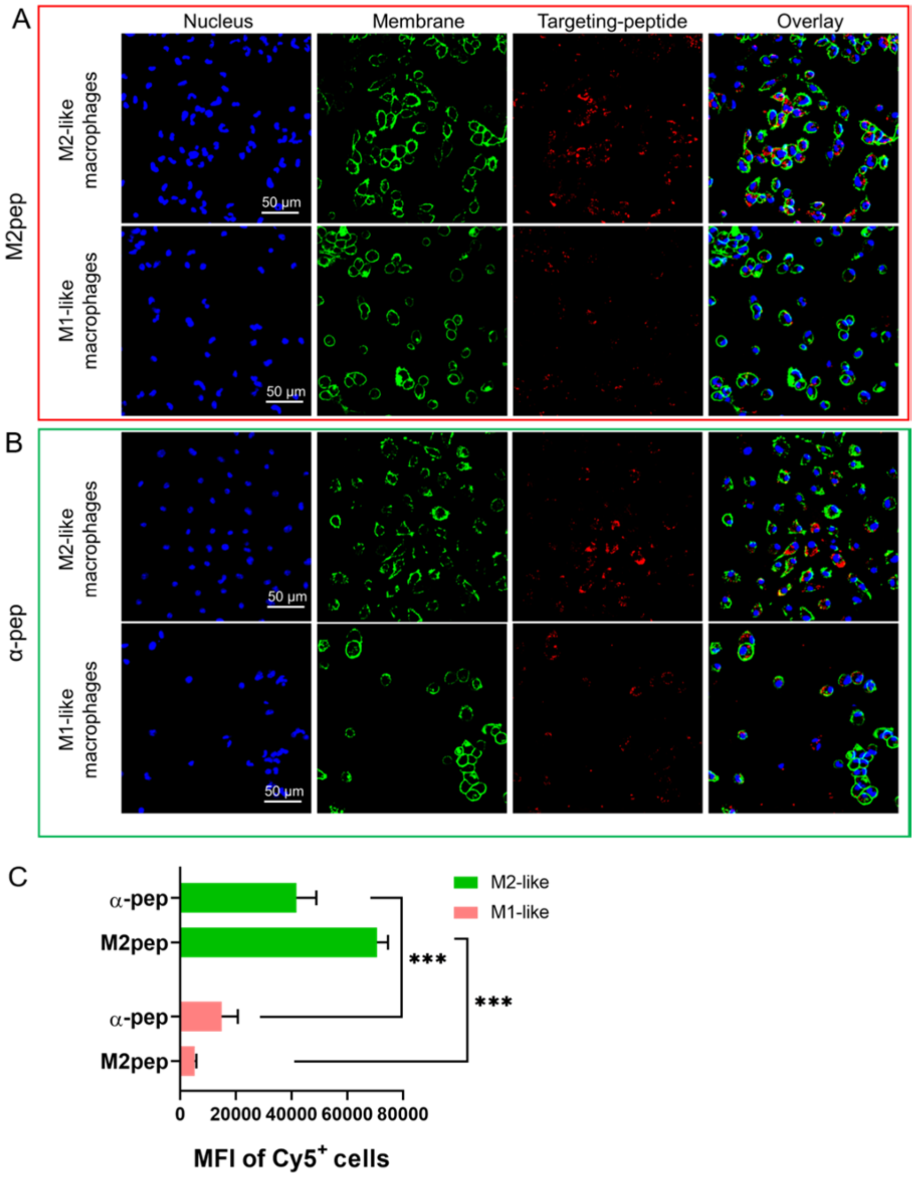

Figure S3. Confocal images of M2pep and α-pep (not on nanoparticles) targeting to M2-like macrophages. (A) M2pep targeting to M2-like/M1-like macrophages. (B) α-pep targeting to M2-like/M1-like macrophages. (C) The quantification for A and B. Data are expressed as the mean ± standard error of the mean (SEM), n = 3. Differences between two groups were tested

using an unpaired, two-tailed Student's t-test. Differences among multiple groups were tested with one-way ANOVA followed by Tukey's multiple comparison. Significant differences between groups are expressed as follows: *P < 0.05, **P < 0.01, or ***P < 0.001.

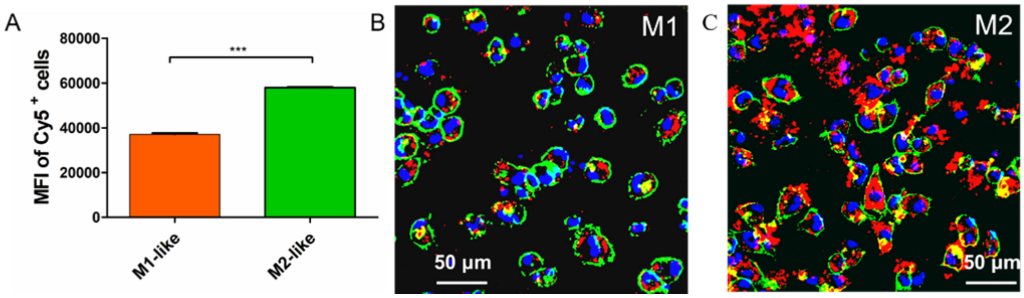

Figure S4. Nano-complexes targeting capacity to M2-like macrophages *in vitro* determined by flow cytometry and confocal imaging. (A) The nano-complexes of uptake by M1-like macrophages and M2-like macrophage cells were analyzed by flow cytometry. (B, C) The nano-complexes (B/H@NPs@CpG-αmp) of uptake by M1-like macrophages and M2-like macrophage cells were analyzed by confocal microscopy. Three independent experiment were analyzed in every group, n = 3. Data are expressed as the mean ± standard error of the mean (SEM). Differences between two groups were tested using an unpaired, two-tailed Student's t-test. Differences among multiple groups were tested with one-way ANOVA followed by Tukey's multiple comparison. Significant differences between groups are expressed as follows: *P < 0.05, **P < 0.01, or ***P < 0.001.

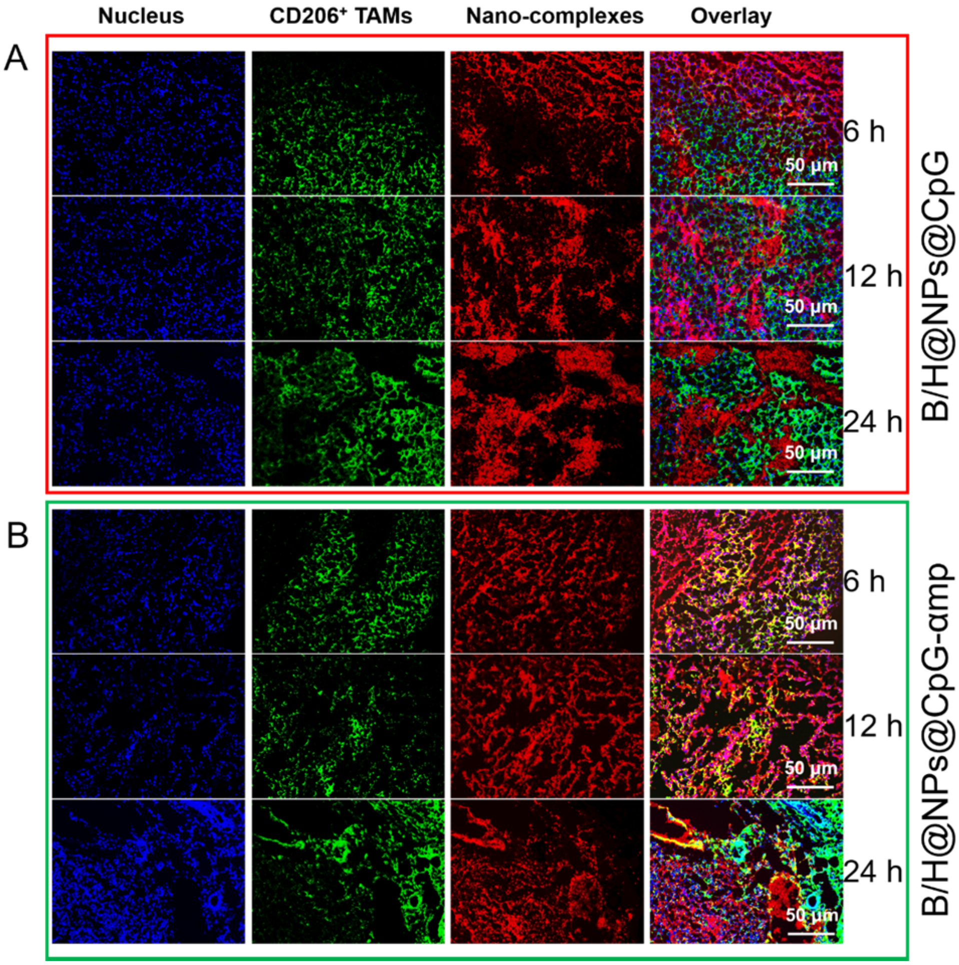

Figure S5. Representative immunofluorescence images for detection of CD206^{+} TAMs targets of B/H@NPs@CpG and B/H@NPs@CpG-αmp nano-complexes at 6 h, 12 h, 24 h after treatment. Yellow represents co-localization of nano-complexes and TAMs. Blue: cell nucleus, red: nano-complex, green: CD206^{+} TAMs. Scale bar: 50 μm. The images were analyzed by automatic multispectral imaging system (PerkinElmer Vectra II).

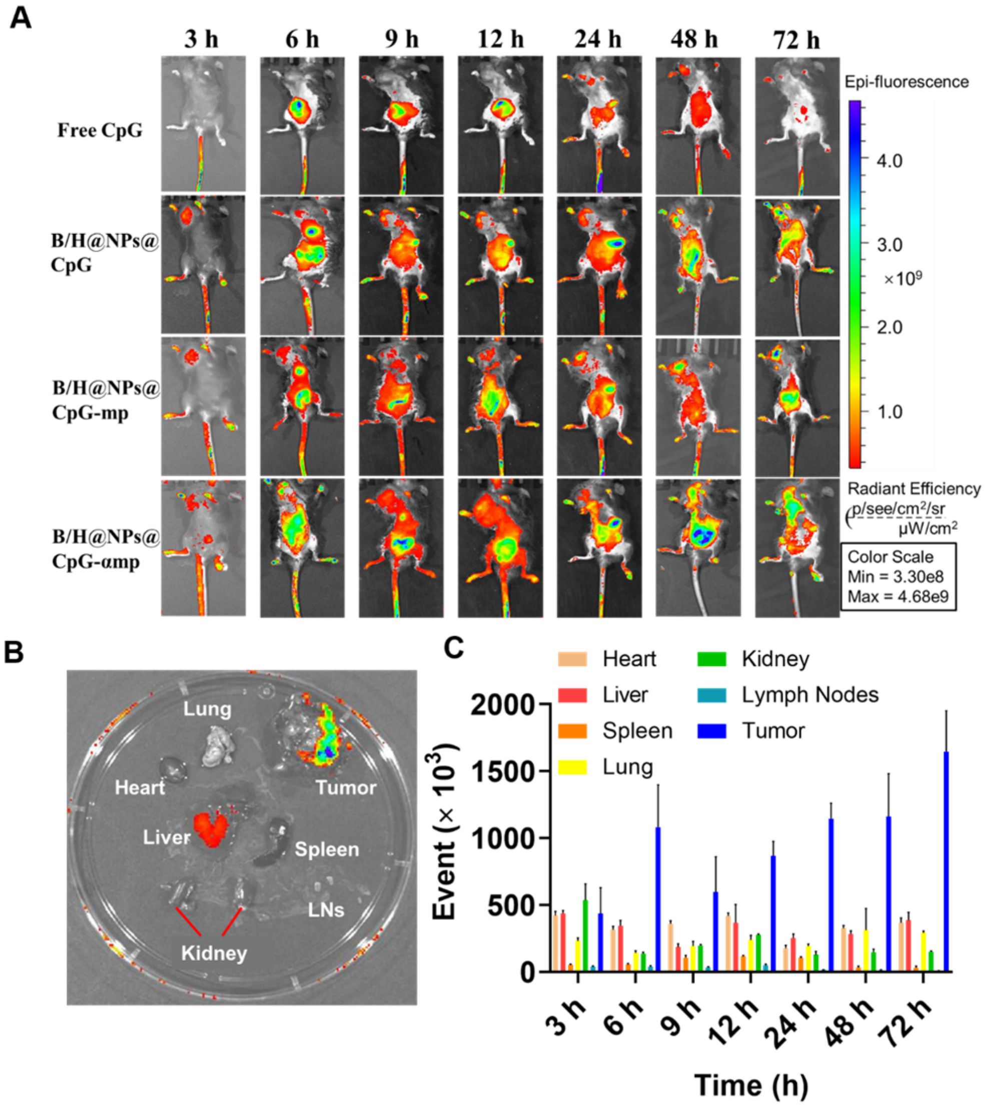

Figure S6. The *in vivo* distributions of the designed nano-complexes. (A) Images of nano-complexes targeting to tumor *in vivo*. (B) The anatomical images of various organs from mice administrated with double-targeted nano-complexes as B/H@NPs@CpG-αmp at 72 h. (C) The dynamic distributions of double-targeted nano-complexes as B/H@NPs@CpG-αmp in various organs were detected by flow cytometry. Three mice were analyzed in every group (n = 3), and one representative image per group is displayed. Data are the mean ± SEM and representative of three independent experiments.

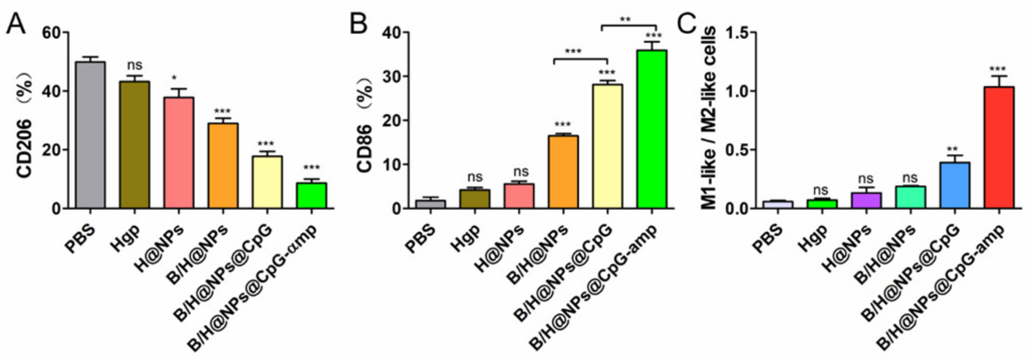

Figure S7. (A, B) The TAMs of M2-like (CD206) and M1-like (CD86) phenotypes was detected by immunofluorescence. (C) The TAMs phenotype of M1-like and M2-like fractions within tumor tissue as represented by CD86 and CD206 biomarkers respectively after treatment with different nano-complexes. The data were analyzed by automatic multispectral imaging system (PerkinElmer Vectra II). Three mice were analyzed in every group (n = 3), and data are the mean ± SEM and representative of three independent experiments. Differences between two groups were tested using an unpaired, two-tailed Student's t-test. Differences among multiple groups were tested with one-way ANOVA followed by Tukey's multiple comparison. Significant differences between groups are expressed as follows: *P < 0.05, **P < 0.01, or ***P < 0.001； The "ns", "*", "**", and "***" are for group *vs* PBS.

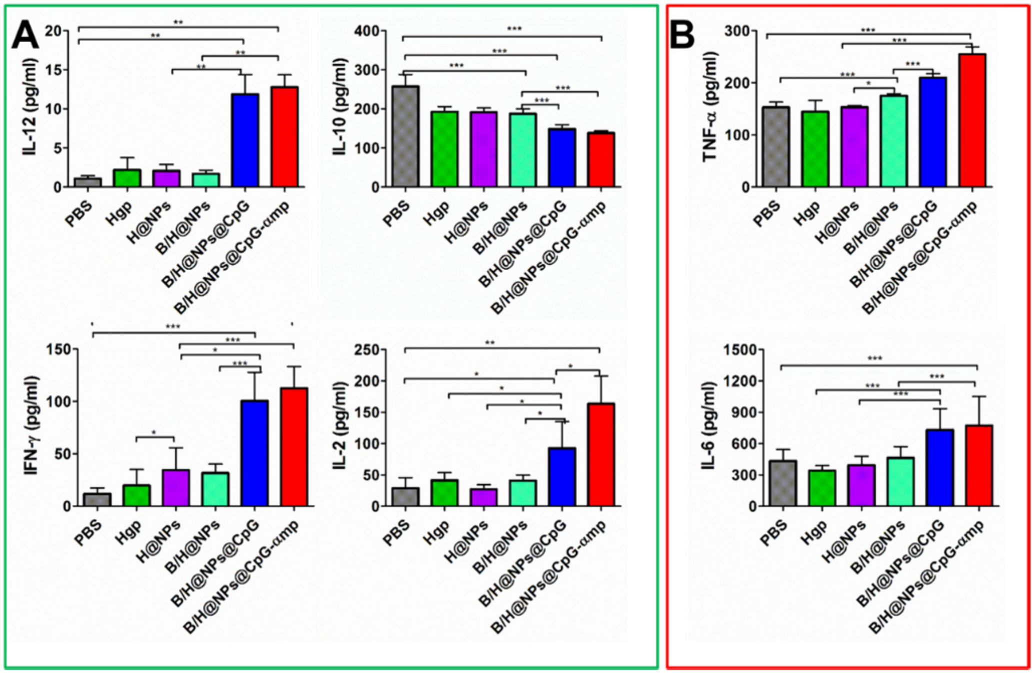

Figure S8. The expression of cytokines at tumor sites of tumor-bearing mice after treatment with different nano-complexes. The expression of IL-12, IL-2, IFN-γ and IL-10 cytokines at the tumor tissue site was analyzed using an ELISA kit (A). The expression of IL-6 and TNF-α in serum of tumor-bearing mice was analyzed using an ELISA kit (B). Data are expressed as the mean ± standard error of the mean (SEM). Differences between two groups were tested using an unpaired,

two-tailed Student's t-test. Differences among multiple groups were tested with one-way ANOVA followed by Tukey's multiple comparison. Significant differences between groups are expressed as follows: *P < 0.05, **P < 0.01, or ***P < 0.001.

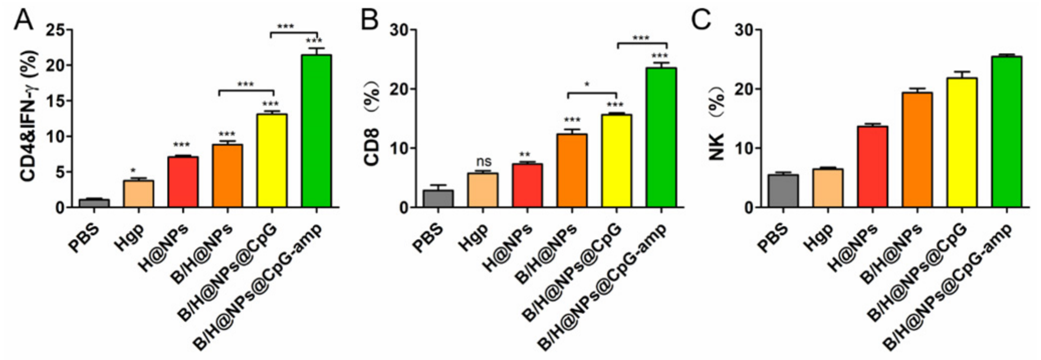

Figure S9. (A, B, C) The infiltration of Th1 ($CD4^+$/IFN-γ), $CD8^+$ and NK cells at tumor sites examined by immunofluorescent staining. The data were analyzed by automatic multispectral imaging system (PerkinElmer Vectra II). Three mice were analyzed in every group (n = 3), and data are the mean ± SEM and representative of three independent experiments. Differences between two groups were tested using an unpaired, two-tailed Student's t-test. Differences among multiple groups were tested with one-way ANOVA followed by Tukey's multiple comparison. Significant differences between groups are expressed as follows: *P < 0.05, **P < 0.01, or ***P < 0.001; the "ns", "*", "**", and "***" are for group *vs* PBS.

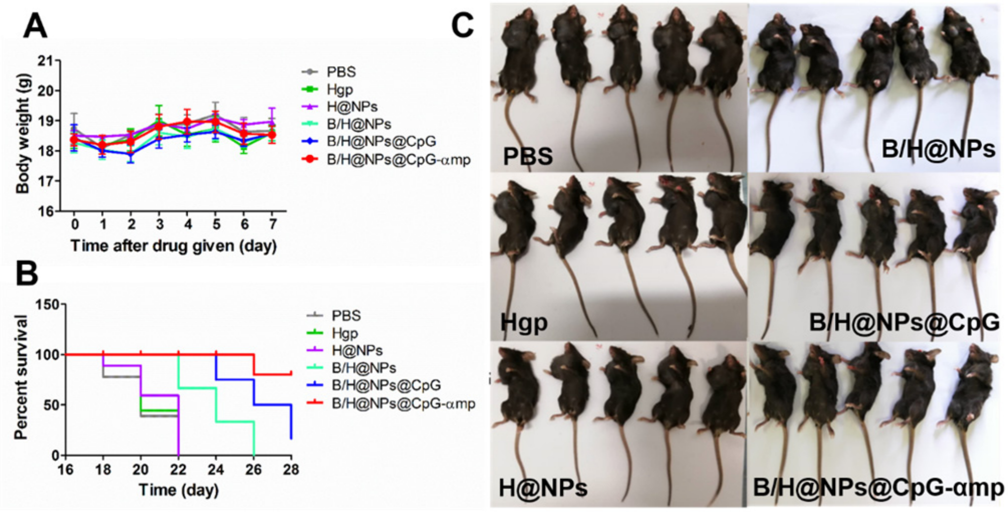

Figure S10. Antitumor responses of various formulations of nano-complexes in the B16 tumor model. The body weight of tumor-bearing mice given daily nano-complex formulations for 7 days of treatment, starting at 8 days post-implantation of tumors (A). The tumor survival curves after treatment with different nano-complex formulations (B). Representative images of tumor-bearing mice in each treatment group (C). Five mice were analyzed in every group (n = 5), and data are the mean ± SEM and representative of five independent experiments.

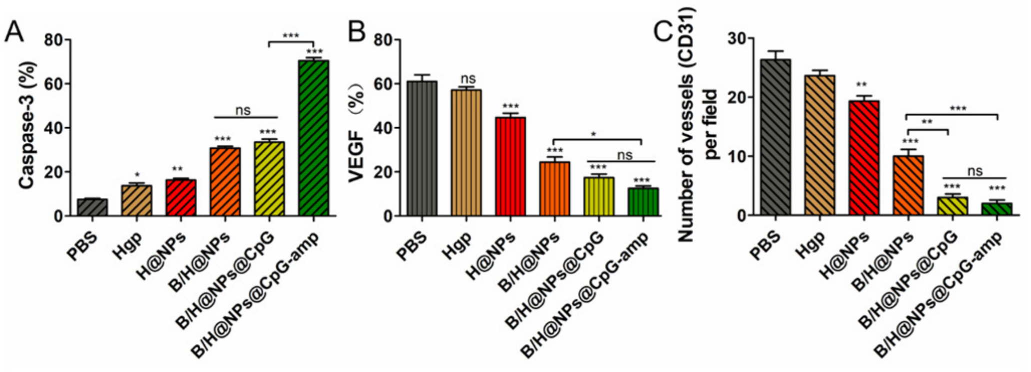

Figure S11. (A) Caspase-3 analysis of tumor tissue indicating apoptotic cells by immunofluorescence in frozen tumor sections. (B) VEGF labeled by immunofluorescence indicating the quality of pro-angiogenesis secretion per field after treatment by different nano-complexes. (C) The number of vessels per field presented by CD31 labeled after treatment by different nano-complexes. The data were analyzed by automatic multispectral imaging system (PerkinElmer Vectra II). Three mice were analyzed in every group (n = 3), and data are the mean ± SEM and representative of three independent experiments. Differences between two groups were tested using an unpaired, two-tailed Student's t-test. Differences among multiple groups were tested with one-way ANOVA followed by Tukey's multiple comparison. Significant differences between groups are expressed as follows: *P < 0.05, **P < 0.01, or ***P < 0.001; The "ns", "*", "**", and "***" are for group *vs* PBS; The "ns", "*", "**", and "***" are for group *vs* PBS.

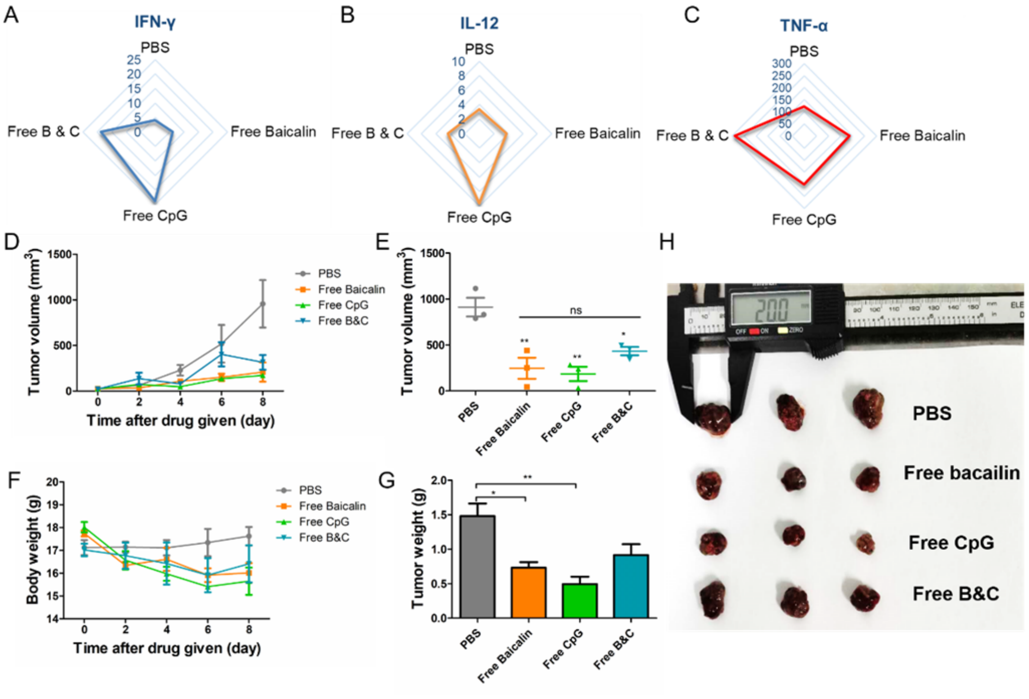

Figure S12. The antitumor effect and immunomodulatory response of free baicalin, free CpG and free baicalin &CpG (Free B&C). (A, B, C) The IFN-γ, IL-12 and TNF-α expression in the

supernatant from B16 tumor tissues was analyzed using an ELISA kit. (D) Tumor volume from mice that received iv. different formulations. (E) Tumor volume was obtained from mice on the last day of the anti-tumor experiment. (F) The body weight curve from tumor-bearing mice after formulations were administered continuously for 7 days, starting at 8 days post-implantation of tumors. (G) The tumor weight change from tumor-bearing mice. (H) Images of B16 tumors harvested from mice in each treatment group. Three mice were analyzed in every group (n = 3). Data are the mean ± SEM and representative of three independent experiments. Differences between two groups were tested using an unpaired, two-tailed Student's t-test. Differences among multiple groups were tested with one-way ANOVA followed by Tukey's multiple comparison. Significant differences between groups are expressed as follows: *P < 0.05, **P < 0.01, or ***P < 0.001; The "ns", "*", "**", and "***" are for group *vs* PBS.

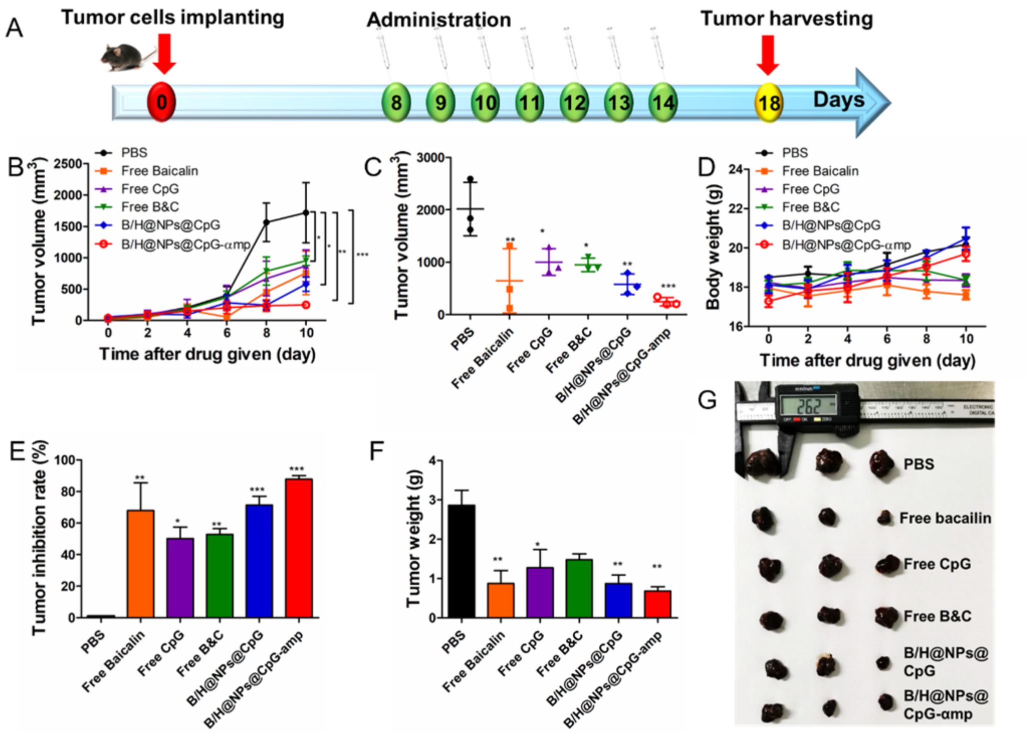

Figure S13. Effective anti-tumor response after treatment with different formulations in the B16F10-bearing mice model. (A) Schematic illustration of administration time sequence for tumor-bearing mice. (B) Tumor volume from mice received iv. different formulations. (C) Tumor volume on Day 18. (D) The body weight of tumor-bearing mice after last treatment with different formulations (from day 8 to 18). (E)Tumor inhibition rates after receiving iv. various formulations. (F) Change in tumor weight for tumor-bearing mice treatment with different formulations. (G) Images of tumors harvested from mice in each treatment group. Three mice were analyzed in every group (n = 3). Data are the mean ± SEM and representative of three independent experiments. Differences between two groups were tested using an unpaired, two-tailed Student's t-test. Differences among multiple groups were tested with one-way ANOVA followed by Tukey's multiple comparison. Significant differences between groups are expressed as follows: *P < 0.05, **P < 0.01, or ***P < 0.001; The "ns", "*", "**", and "***" are for groups *vs* PBS.

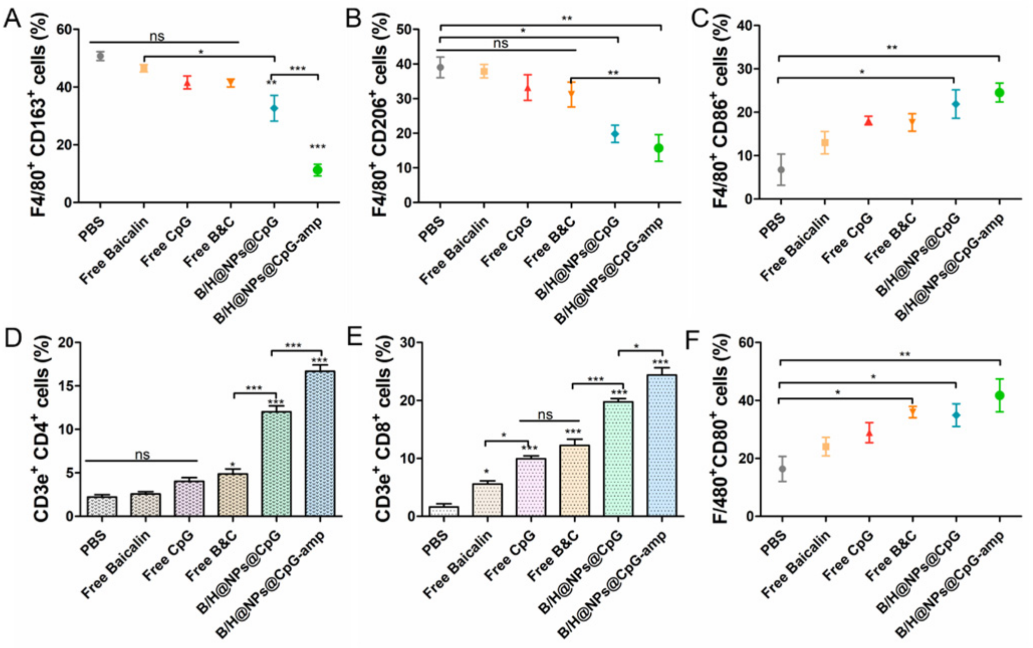

Figure S14. Tumor microenvironments remodeling after treatment with different nano-complexes in B16F10-beared mice model. The TAMs phenotype reversion at tumor site after treatment by different formulations after iv. in vivo. The M2-like TAMs surface markers as CD163 (A) and CD206 (B), and the M1-like surface markers as CD86 (C) and CD80 (F) on TAMs were analyzed by flow cytometry. (D, E) The infiltration of Th1 (CD4+) and CTL (CD8+) cells at tumor sites was examined by flow cytometry. Three mice were analyzed in every group (n = 3), and one representative images per group are display. Data are the mean ± SEM and representative of three independent experiments. Differences between two groups were tested using an unpaired, two-tailed Student's t-test. Differences among multiple groups were tested with one-way ANOVA followed by Tukey's multiple comparison. Significant differences between groups are expressed as follows: *P < 0.05, **P < 0.01, or ***P < 0.001; The "ns", "*", "**", and "***" are for group *vs* PBS.

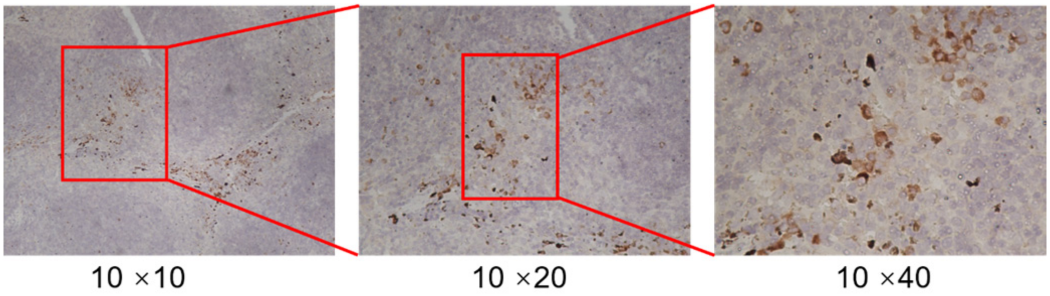

Figure S15. Immunohistochemical sections of spleen tissue from tumor-bearing mice. The antibody HMB-45 was used for melanoma staining. Three mice were analyzed in this study (n = 3), and one representative image is displayed.

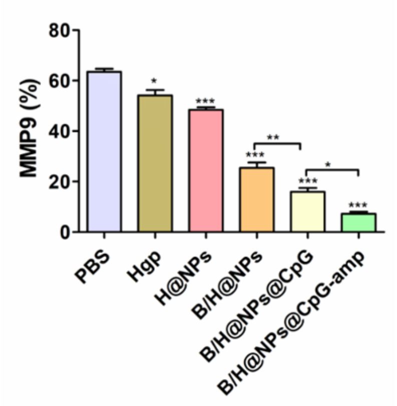

Figure S16. Matrix metalloproteinases (MMPs) associated with metastasis in tumor sites were analyzed by immunofluorescence staining of MMP9. The data were analyzed by automatic multispectral imaging system (PerkinElmer Vectra II). Three mice were analyzed in every group (n = 3), and data are the mean ± SEM and representative of three independent experiments. Differences between two groups were tested using an unpaired, two-tailed Student's t-test. Differences among multiple groups were tested with one-way ANOVA followed by Tukey's multiple comparison. Significant differences between groups are expressed as follows: *P < 0.05, **P < 0.01, or ***P < 0.001; The "ns", "*", "**", and "***" are for group *vs* PBS.

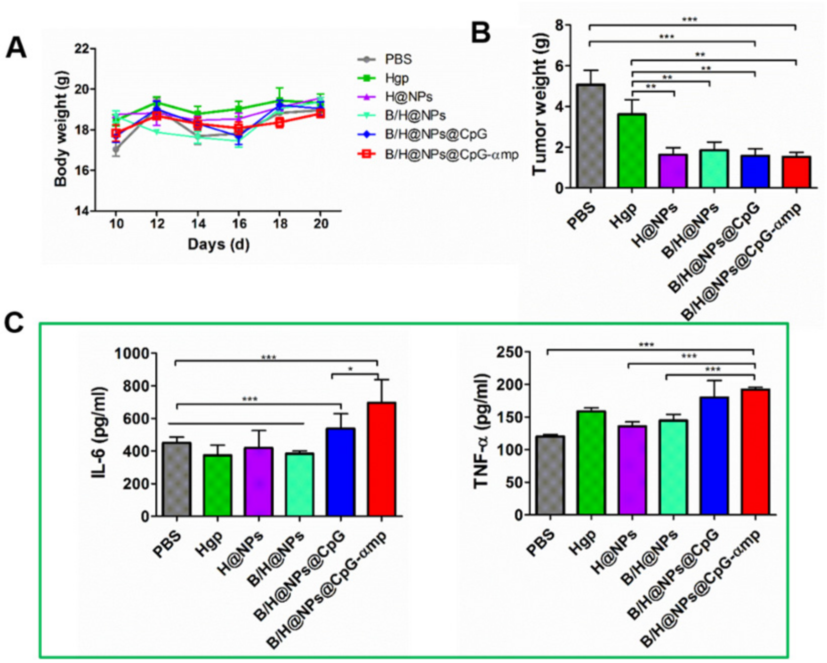

Figure S17. Tumor inhibition responses measured after receiving *i.v.* injections of nano-complex formulations in macrophage priming, preventative experiments. The body weight curve from tumor-bearing mice after nano-complexes were administered daily for 7 days (A). The change of tumor weight from mice receiving *i.v.* injections of various nano-complex (B). The expression of IL-6 and TNF-α in the serum of tumor-bearing mice were analyzed using an ELISA kit (C). Five mice were analyzed in every group (n = 5), and data are the mean ± SEM and representative of five independent experiments. Differences between two groups were tested using an unpaired,

two-tailed Student's t-test. Differences among multiple groups were tested with one-way ANOVA followed by Tukey's multiple comparison. Significant differences between groups are expressed as follows: *P < 0.05, **P < 0.01, or ***P < 0.001.

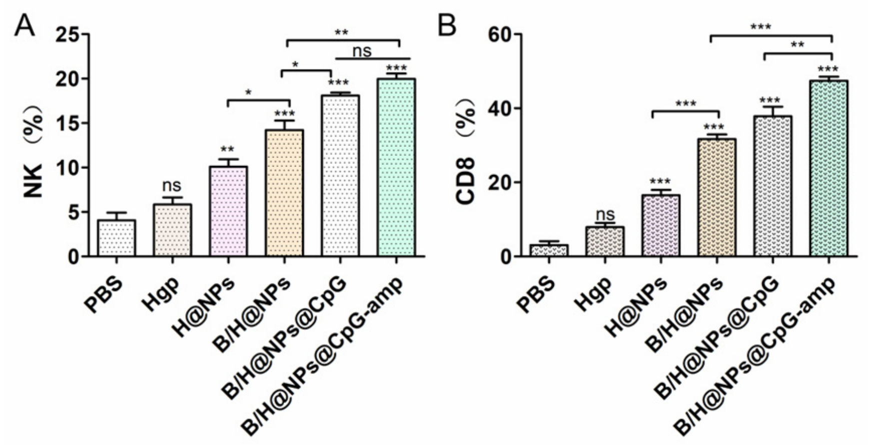

Figure S18. (A, B) The infiltration of $CD8^+$ T cells and NK cells at tumor sites after preventive treatment was examined by immunofluorescent staining. The data were analyzed by automatic multispectral imaging system (PerkinElmer Vectra II). Three mice were analyzed in every group (n = 3), and data are the mean ± SEM and representative of three independent experiments. Differences between two groups were tested using an unpaired, two-tailed Student's t-test. Differences among multiple groups were tested with one-way ANOVA followed by Tukey's multiple comparison. Significant differences between groups are expressed as follows: *P < 0.05, **P < 0.01, or ***P < 0.001; The "ns", "*", "**", and "***" are for group *vs* PBS.

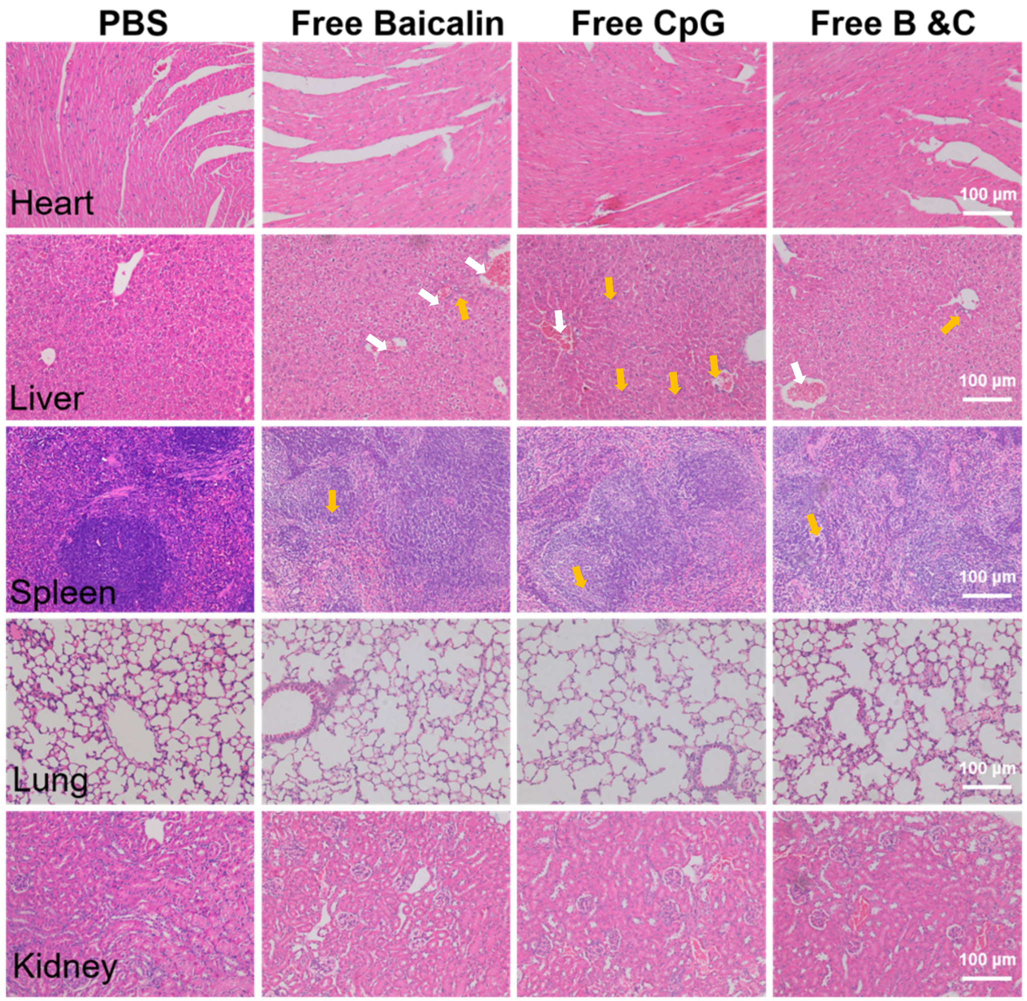

Figure S19. *In vivo* toxicity of formulations of Free Baicalin, CpG and Free B&C. H&E-stained slice images of major organs from the different groups.
